# Molecular Mechanisms of HIV Protease Inhibitors Against HPV-Associated Cervical Cancer: Restoration of *TP53* Tumour Suppressor Activities

**DOI:** 10.3389/fmolb.2022.875208

**Published:** 2022-05-10

**Authors:** Lilian Makgoo, Salerwe Mosebi, Zukile Mbita

**Affiliations:** ^1^ Department of Biochemistry, Microbiology and Biotechnology, University of Limpopo, Sovenga, South Africa; ^2^ Department of Life and Consumer Sciences, University of South Africa, Florida, South Africa

**Keywords:** cervical cancer, human papilloma virus, p53, HIV protease inhibitors, E6, anti-HIV drugs, PDZ proteins

## Abstract

Cervical cancer is a Human Papilloma virus-related disease, which is on the rise in a number of countries, globally. Two essential oncogenes, *E6* and *E7*, drive cell transformation and cancer development. These two oncoproteins target two of the most important tumour suppressors, p53 and pRB, for degradation through the ubiquitin ligase pathway, thus, blocking apoptosis activation and deregulation of cell cycle. This pathway can be exploited for anticancer therapeutic interventions, and Human Immunodeficiency Virus Protease Inhibitors (HIV-PIs) have attracted a lot of attention for this anticancer drug development. HIV-PIs have proven effective in treating HPV-positive cervical cancers and shown to restore impaired or deregulated p53 in HPV-associated cervical cancers by inhibiting the 26S proteasome. This review will evaluate the role players, such as HPV oncoproteins involved cervical cancer development and how they are targeted in HIV protease inhibitors-induced p53 restoration in cervical cancer. This review also covers the therapeutic potential of HIV protease inhibitors and molecular mechanisms behind the HIV protease inhibitors-induced p53-dependent anticancer activities against cervical cancer.

## Introduction

Besides the current SARS-CoV-2 virus-related pandemic, cancer remains an alarming threat to human kind, not only in South Africa, but globally. Cancer, a cluster of neoplastic disorders caused by deregulated cell growth, is due to mutations in key regulatory genes, resulting in deregulated cell proliferation, resistance to apoptosis, and amplified cell survival signals (Sinha, 2018). According to Sung et al. (2021), cancer is one of the leading causes of death worldwide, contributing to an estimated 10 million deaths by 2020. In 2018, cancer claimed 9.6 million lives, 70% of which occurred in low and middle income countries (Dalton et al., 2019). The rising burden of cancer in low and middle-income countries is attributed to aging populations, lifestyle changes and adoption of Western habits, which include smoking and alcohol consumption (List and O’Connor, 2020). Furthermore, there are over 200 different types of cancers with different metastatic nature and unique characteristics, making the fight against cancer far more complex. Therefore, cancer remains a major public health concern exerting enormous pressure on healthcare systems. Additionally, oncoviruses play crucial roles in the carcinogenesis process, leading to the manifestation of different types of cancers, including cervical cancer (Bray et al., 2018).

**FIGURE 1:**
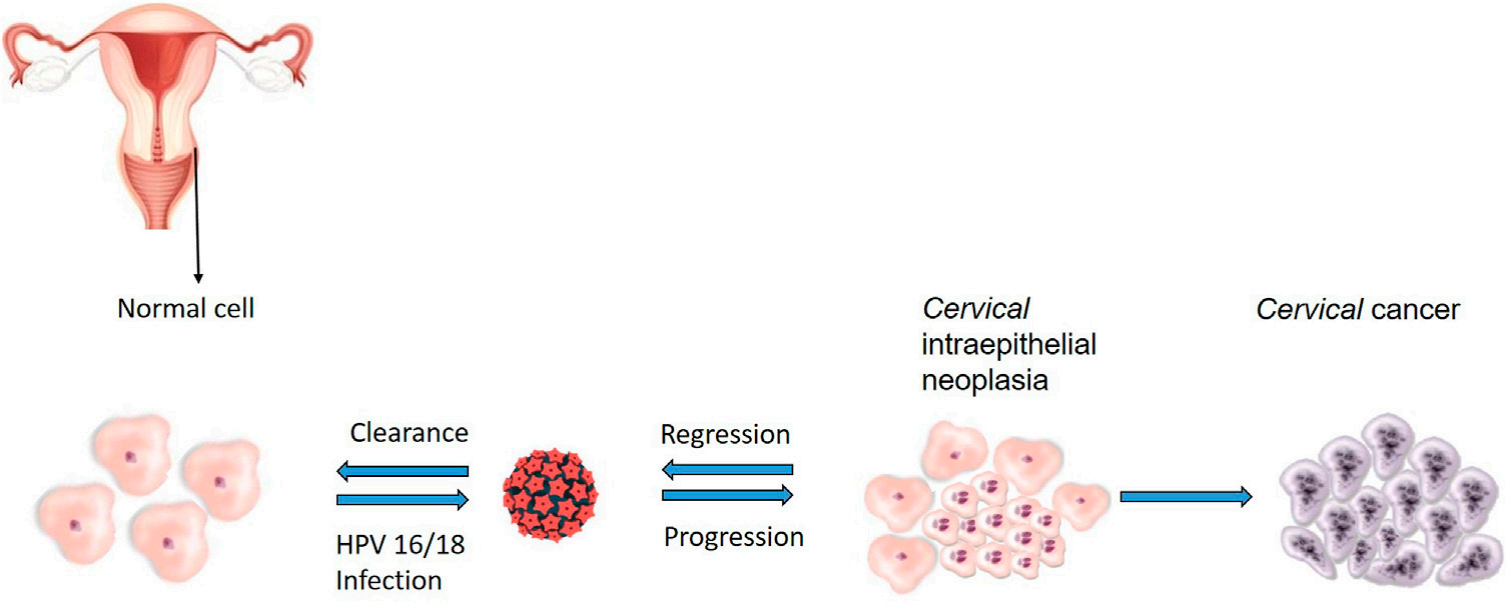
Sequence of events leading to the development of HPV-related cervical cancer. Cervical intraepithelial neoplasia develop when squamous cells grow abnormally after persistent HPV infection, most of these changes resolve over time but in others, the integration of viral particles transforms cells and enables them to progress to the development cervical cancer.

Cervical cancer is the second most common cancer among women in South Africa, and an estimated 5,743 new cases and over 3,000 deaths are reported annually (Akokuwebe et al., 2021). Globally, cervical cancer is the fourth most diagnosed cancer in women with 342,000 deaths recorded in 2020 (Sung et al., 2021). Cancer of the cervix is characterized by abnormal proliferation of glandular or squamous cells lining the cervix, mostly due to infection with high risk Human papillomavirus (HPV) (Jalil and Karevskiy, 2020). Biological and epidemiological evidence exists, which confirmed HPV, a sexually transmitted virus, as a primary causative agent of the pre-cancerous lesions that lead to full blown invasive cervical cancer (Li et al., 2011; Guan et al., 2012; de Martel et al., 2017; Jalil and Karevskiy, 2020). As demonstrated in Figure 1, pre-cancerous changes can occur in cervix cells infected with high-risk HPV, and most of these changes resolve over time, but some do progress to cervical cancer. HPV is responsible for almost 100% of cervical cancers (de Martel et al., 2017).

The genome of HPV encodes gynaecological cancer-related oncogenes, which include E5, E6 and E7 (Jiang and Yue, 2014). These oncogenes encode proteins that are implicated in carcinogenesis, a process that has been demonstrated in both *in vitro* and *in vivo* models (Hoppe-Seyler et al., 2018). The HPV prevents apoptosis in E6 expressing cells by inactivating tumour suppressors, such as p53 (Jiang and Yue, 2014). E5 protein protects cells from apoptosis triggered either by tumour necrosis factor-related apoptosis-inducing ligand (TRAIL) or by Fas ligand (FasL). This is achieved through downregulating the Fas receptor and impairing the assembly of TRAIL-induced death-inducing signalling complex (DISC) (Kabsch and Alonso, 2002). On the other hand, E6 oncoprotein predominantly inhibits apoptosis by regulating p53 turnover (Hengstermann et al., 2001; Murray-Zmijewski et al., 2008; Abboodi et al., 2021), thus inhibiting p53-mediated apoptosis pathways; and lastly, E7 oncoprotein is involved in both apoptosis activation and inhibition by targeting pRb (McLaughlin-Drubin and Münger, 2009; Zimmermann et al., 2011). Clearly, these HPV proteins function by targeting key pathways that are necessary for sustaining cellular homeostasis. The most studied and well-understood HPV oncoproteins, E6 and E7, aim their effect on deregulating cell survival signalling pathways, thus, promoting viral replication; however, these effects also upset cell homeostasis, consequently leading to carcinogenesis.

Most of HPV infections are asymptomatic and partners can unknowingly pass it on to one another through sexual contact (Botha and Dochez, 2012). Majority of HPV-related cancers are prevented by using commercial HPV vaccines; however, these vaccines are ineffective in eradicating persistent HPV infections, and have not been demonstrated to slow down the HPV-related progression of malignant tumours (Cheng et al., 2020). Although cervical cancer can be prevented and managed at its early stages, most women continue to lose their lives due to ineffective pre-cancer screening and treatment programs, especially in developing countries, which include South Africa (SA).

Studies have shown that through protein-protein interactions, HPV affect the functioning of different cellular proteins, which include pRB, p53 and PDZ-containing proteins (Nagasaka et al., 2013; Wang et al., 2017). HPV targets cellular proteins containing the PDZ (PSD-95/Dlg-A/ZO-1) domain, and these proteins are involved in cell signalling, epithelial polarity and membrane trafficking (Nagasaka et al., 2013). Examples of PDZ proteins regulated by HPV in cervical cancer include the Na(+)/H(+) exchange regulatory factor 2 (NHERF-2) (Saidu et al., 2019), a tumour suppressor that regulates endothelial proliferation. To regulate NHERF-2, E6 oncoproteins of high risk HPV bind to it and target it for proteasome-mediated degradation (Saidu et al., 2019). Drugs such HIV protease inhibitors have been shown to disturb HPV interactions with proteins involved in cell cycle and apoptosis such as p53 (Park et al., 2021), however, there is no information on the effect of HIV protease inhibitors on HPV-PDZ protein interactions.

To promote cervical cancer pathogenesis, HPV interrupts cellular regulatory machinery by inducing the expression of viral proteins, which promote cellular transformation by modulating the expression p53 (Cobzeanu et al., 2019). Since p53 expression varies according to the stage of cervical cancer (Pandey et al., 2016), identifying new role players in the regulation of p53 is pivotal. One of these key role players are the HIV protease inhibitors, which have been implicated in enhancing the expression of p53, thus, inhibiting the progression of cervical cancer (Xia et al., 2017). Under normal cell homeostasis, p53 regulates the strict compliance of individual cells to maintain cell homeostasis, thus impaired p53 leads to cancer (Mehta et al., 2021).

### Epidemiology of Human Papillomavirus Infection

The Human Papillomaviruses are double-stranded DNA viruses characterised by circular DNA with no envelope (Yilmaz et al., 2018). HPV infects humans and different animal species; zur Hausen, 1977 revealed that HPV is the main cause of cervical cancer. Since HPV is sexually transmissible, it was previously estimated that about half of sexually active adults would contract HPV in their lifetime (Chesson et al., 2014). HPVs are classified into five different major genera, which include alpha (*α*), beta (*β*), gamma (*γ*), mu (*µ*), and nu (*ν*) (De Villiers et al., 2004; Brancaccio et al., 2018). Amongst these HPV genera, *α*, *β* and *γ* are implicated in the development of different diseases, including cervical cancer (alpha), skin cancer (beta) and head and neck squamous cell carcinoma (gamma) (De Villiers et al., 2004; Agalliu et al., 2016; Tommasino, 2017; Mirbahari and Sadeghi, 2018; Becerril et al., 2021). HPV is most prevalent among adolescents and young adults between ages 15 and 25 and declines significantly after this age (Baussano et al., 2013). Alpha, beta and gamma HPV genera are most implicated in this age groups (Sias et al., 2019). HPVs can be categorised into high-risk and low-risk, with the former mostly associated with carcinogenesis, and these include HPV 16 and 18, while the latter are barely linked to carcinogenesis, and are exemplified by HPV 6 and 11 (Li et al., 2011; Guan et al., 2012; Cornall et al., 2013). High-risk HPVs account for 5% of all cancers, worldwide, with cervical cancer being the most prevalent (de Martel et al., 2017; de Sanjosé et al., 2018).

### HPV Oncoproteins Promote p53 Proteasome Degradation

**FIGURE 2:**
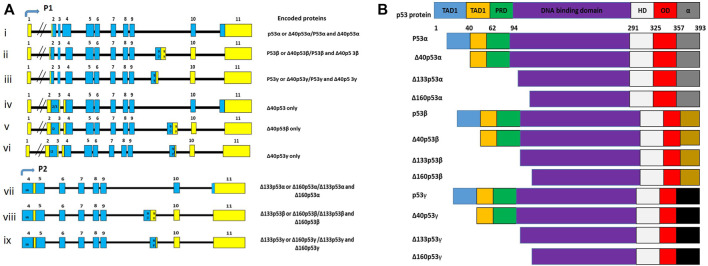
**(A)** *TP53* mRNA transcripts, coding exons (blue), noncoding exons (yellow) and alternative exons (i). Although there are 15 transcripts encoded in the *TP53* gene, only 9 transcripts are often presented because some code for the same protein isoforms. **(B)** There are 12 different p53 isoforms, containing various combination of the different domains, which include the transactivation domain 1 (TAD1), the transactivation domain 2 (TAD2), the DNA binding domain (DBD), the proline-rich domain (PRD), the oligomerization domain (OD), the hinge domain (HD), and the negative regulation domain (α). Adapted from Anbarasan and Bourdon (2019).

The TP53 is a tumour suppressor gene, which is localised on chromosome 17p13.1, and is transcribed into 15 transcripts due to the use of alternative promoters (P1 and P2), alternative splicing (Δ40p53β and Δ40p53γ) and alternative initiation sites of translation (Δ40p53, Δ133p53α, Δ133p53β, and Δ133p53γ) (Marcel and Hainaut, 2009; Khoury and Bourdon, 2011; Joruiz and Bourdon, 2016). These transcripts are translated into 12 different protein isoforms, which differ by domain arrangements (Figure 2). The findings from clinical studies suggest that the expression patterns of specific p53 isoforms could predict tumour progression, clinical response, and prognosis (Kim and An, 2016). Since *TP53* variants are implicated in cancer, it is possible that HPV modulate these variants to inhibit and degrade p53 in order to abolish its cancer prevention effects (Sousa et al., 2011). Although studies on the interaction between p53 isoforms and HPV are limited, research in this field will contribute significantly to finding specific HPV-associated cancer biomarkers for diagnostic and therapeutic purpose.

**FIGURE 3:**
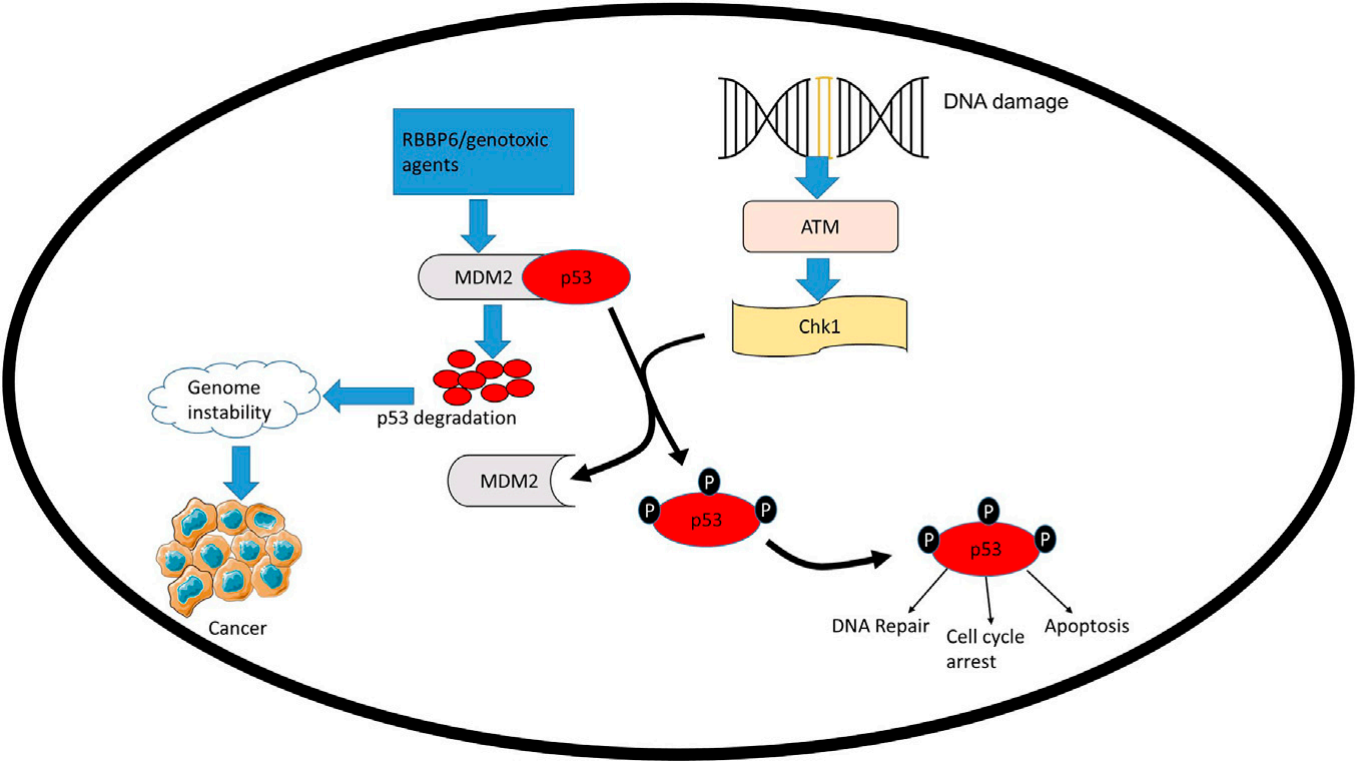
MDM2 regulates the stability of TP53*,* thus, influencing its role in cell homeostasis. Negative regulators of TP53 such as RBBP6 can interact with MDM2 and facilitate the ubiquitination and degradation of TP53 by MDM2. Overexpression of MDM2 significantly delays DNA repair, resulting in damage persisting for longer thus promoting genome instability, which leads to cancer development.

TP53 mutations mostly occur in its DNA-binding domain coding region, interfering with p53’s ability to bind to DNA and transactivate downstream genes, thus promoting different cancers, including cervical cancer (Kato et al., 2003; Banister et al., 2017; He et al., 2019a). The DNA-binding domain is not only affected by mutations, it is also implicated in the interactions between HPV oncoproteins and p53 (Bernard et al., 2011). In response to various stress signals (such as DNA damage, oncogene activation, hypoxia, and nutrient depletion), p53 is stabilized and activated by post-translational modifications such as the activation of ataxia telangiectasia mutated (ATM) (Parrales and Iwakuma, 2015). p53 then binds to p53 response elements on different genes, either activating DNA damage, cell cycle, or apoptosis (Figure 3). Binding of p53 to these genes triggers their expression, thus, regulating cell cycle, apoptosis, autophagy and DNA repair. The effect of p53 interaction with these genes is summarized in Table 1. By activating genes, such as Bax, CDKN1A and DDB21, p53 is able to orchestrate a variety of mechanisms; namely, apoptosis, cell cycle and DNA-damage-response mechanisms to avoid cancer development (Table 1).

**TABLE 1:** Genes transcriptionally activated by p53 and their functions.

Genes activated by p53	Function	References
DDB21	DNA repair	Eischen (2016); Hafner et al. (2019)
XPC1	DNA repair	Eischen (2016); Hafner et al. (2019)
RRM2B	DNA repair	Tanaka et al. (2000); Chae et al. (2016)
BAX	Apoptosis	Chipuk and Green (2005); Aubrey et al. (2018)
APAF1	Apoptosis	Purvis et al. (2012); Feroz and Sheikh (2020)
DRAM1	Autophagy	Crighton et al. (2006); Broz et al. (2013)
ULK1	Autophagy	Gao et al. (2011)
CDKN1A	Cell cycle arrest	Hafner et al. (2019)
GADD45a	Cell cycle arrest	Han et al. (2019)

**FIGURE 4:**
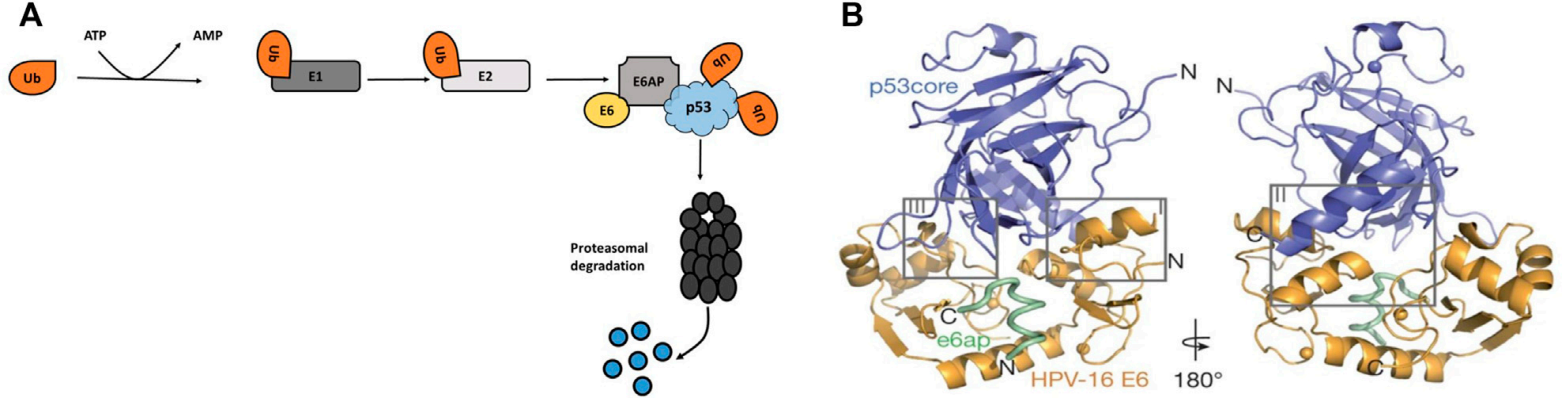
**(A)** HPV oncoprotein E6 induces p53 proteasome degradation through E6AP. A cascade of ubiquitination occurs when E1 protein activate ubiquitin, which allows E2 conjugating enzymes to transfer the ubiquitin to E6AP, a ubiquitin-protein ligase. **(B)** Structural interaction between the HPV-16 E6, E6AP and p53 protein (Martinez-Zapien et al., 2016). E6 interact with p53 by binding to the LxxLL motif of E6AP, the LxxLL motif is sufficient to render E6 able to promote p53 degradation.

E6 oncoproteins of high-risk HPVs interfere with the transcriptional activity of p53. Studies showed that E6 promote p53 degradation in the presence of E6-associated protein (E6AP) (Figure 4A) (Talis et al., 1998; Li et al., 2019). It is not only the HPV that targets p53 for degradation to subdue its tumour suppressive effects (Travé and Zanier, 2016), but other proteins regulate p53 through the 26S proteasome and ubiquitination pathway. These include Retinoblastoma Binding Protein 6 (RBBP6) (Li et al., 2007), Tripartite motif protein 25 (TRIM25) (Zhang et al., 2015), F-box and WD repeat domain-containing 7α (FXBW7α) (Tripathi et al., 2019), and the list will continue lengthening as more studies emerge towards understanding the role of p53.

Martinez-Zapien et al. (2016) demonstrated the interactions between p53 and HPV-16 oncoprotein, E6 (Figure 4B). The structure showed that the interaction is governed by the LxxLL (L is leucine and X is any amino acid) motif of the E6AP. In the cellular ubiquitin ligase E6AP, E6 binds to a short consensus sequence in the LxxLL motif, and the LxxLL motif is sufficient to render E6 liable to interact with p53. Bernard et al. (2011) also showed that the core DNA binding domain of p53 is required for the interaction with E6/E6AP. Since p53 is mostly mutated in cancerous cells, especially in its DNA-binding domain, understanding the E6-mediated degradation of the p53 mutants is vital. Interestingly, Bernard et al. (2011) found that unlike the wild type p53, mutant p53 was resistant to HPV16 E6-mediated degradation. Li et al. (2019) showed similar effects on the mutant p53 where the HPV E6 failed to promote the degradation of a mutant p53; the study highlighted that it is possible to re-establish the tumour suppressor function of wild type p53 by using a p53 mutant. However, earlier findings indicated that p53 mutations occur rarely in HPV-associated cervical cancers (Denk et al., 2001) and these mutations are more common in HPV-negative tumours than HPV-positive tumours (Banister et al., 2017).

E6 oncoprotein also interacts with other cellular proteins which are linked to p53, stimulating their ubiquitination and their subsequent degradation (White et al., 2012; Mesri et al., 2014). These proteins include tumour suppressor, p300, which is implicated in p53 acetylation and activation (Xie et al., 2014), and Bak protein, which is involved in apoptosis induction (Underbrink et al., 2008). These results show the importance of targeting E6-mediated mechanisms for anticancer therapeutic interventions. A number of compounds, which include arsenic trioxide (Wen et al., 2012) and rutin (Pandey et al., 2021), demonstrated therapeutic potential for the treatment of HPV-associated cervical cancer. This is due to their capacity to downregulate the expression of E6 and E7 proteins, thus restoring p53 expression and function (Wen et al., 2012; Pandey et al., 2021). Identifying new drugs to disturb p53 and HPV-16 E6 interactions can be a promising strategy to eradicate HPV-associated cancer; some of these drugs include anti-HIV drugs because they have demonstrated anticancer properties (Chow et al., 2009; Subeha and Telleria, 2020). Anti-HIV drugs are also implicated in resolving deregulated cell cycle arrest and resistance to apoptosis during carcinogenesis (Maksimovic-Ivanic et al., 2017; Marima et al., 2020). The antitumour effect of anti-HIV drugs have been shown in a wide range of cancer types including skin cancer (Paskas et al., 2019), lymphoma (Kariya et al., 2014), glioblastoma (Gratton et al., 2018), thyroid cancer (Kushchayeva et al., 2014), Kaposi’s sarcoma (Gantt et al., 2011) and cervical cancer (Qiu et al., 2020).

### The Effect of anti-HIV Drugs on HPV Infection

Cancer is a non-communicable disease but communicable oncogenic viruses, such as Human immunodeficiency virus (HIV) (Hernández-Ramírez et al., 2017; Shiels and Engels, 2017; Stelzle et al., 2021),Epstein-Barr virus (EBV) (Cameron et al., 2018), HPV (De Martel et al., 2017) and Hepatitis B virus (HBV) (Feng et al., 2021) drive the rate of certain cancers, including cervical cancer (Hernández-Ramírez et al., 2017; Shiels and Engels, 2017). The launch of highly active antiretroviral therapy (HAART) has significantly decreased mortality rates associated with HIV, and has improved life expectancy of HIV/AIDS patients (Shiels and Engels, 2017). Despite the use of HAART, HIV patients are still vulnerable to life threatening diseases such as virus-induced cancers (Shmakova et al., 2020).

Anti-HIV drugs have a controversial impact on the natural history of squamous intraepithelial lesions (SIL), the true effect of HAART on HPV is poorly understood because researchers tackle this topic using different study designs, outcomes, and timing of HAART (Blitz et al., 2013; Kelly et al., 2018). In the early days of HAART, Heard et al. (1998) found a non-significant reduction in SIL cases when compared with women who did not receive the treatment. Other reports suggested that HAART has a minimal impact on the incidence of cervical lesions (Heard et al., 2006; Adler et al., 2012). On the other hand, other studies argued by showing that long-term use of HAART is associated with reduced HPV persistence in high-grade cervical intraepithelial neoplasia (Shrestha et al., 2010; Kelly et al., 2017). Additionally, there has been some evidence that non-HPV 16 genotypes have a greater relative prevalence in women with severely compromised immune systems as well as in those without access to HAART (Sahasrabuddhe et al., 2007; McKenzie et al., 2010), further suggesting that HAART duration has a positive impact on reducing the prevalence of other HR-HPVs. Although the effect of HAART on HPV infection is inconclusive, HIV protease inhibitors as one of the components that makes up HAART have shown interesting antitumour effects against HPV associated cervical cancer (Lin et al., 2021; Park et al., 2021).

### Therapeutic Potential of HIV Protease Inhibitors

Since the discovery of HIV-PIs in the early 1990s, HIV-PIs have been used as components of combination antiretroviral regimens to control HIV viral load in people living with HIV (Roberts et al., 1990; Voshavar, 2019). HIV-PIs are required in the final stage of viral life cycle to block the activity of HIV protease enzyme, which is utilised by HIV to split precursors of gag and gag-pol polyproteins required to produce infectious viruses (Yang et al., 2012; Lv et al., 2015). For more than 20 years, HIV-PIs have been the gold standard as therapeutic agents that inhibit HIV protease. Saquinavir was the first HIV-PI to be developed and used as HIV suppressive drug (Vella, 1994). Currently, there are 10 HIV-PIs approved by the FDA, namely, saquinavir, nelfinavir, indinavir, darunavir, amprenavir, atazanavir, fosamprenavir, lopinavir, tipranavir and ritonavir (Lv et al., 2015). The FDA-approved HIV protease inhibitors share similar binding pattern and structural similarities, suggesting that they bind to the HIV protease in a similar manner (Lv et al., 2015). In addition to their antiretroviral properties, HIV-PIs have pleiotropic pharmacological effects, including anticancer effects (Chow et al., 2009; Toschi et al., 2011). As anticancer drugs, HIV-PIs have generally low potency, requiring concentrations above 10 μM to exert anticancer effect (Bernstein and Dennis, 2008), but in both preclinical and clinical trials, chemotherapeutic drugs are being tested in combination with HIV-PIs (Sombogaard et al., 2018) to determine whether the combination of cancer chemotherapy and HAART can improve response rates compared to antineoplastic therapy alone.

HIV-PIs possess direct antitumour properties independent of their antiviral properties, and this was shown in several independent studies, which demonstrated that HIV-PIs exhibited antitumor and antiangiogenic effects independent of viral load and CD4 cell count (Barillari et al., 2003; Monini et al., 2003; Sgadari et al., 2003; Sgadari et al., 2011; Patton et al., 2013). The direct antitumour effects of HIV-PIs have been shown in cervical cancer (Barillari et al., 2012; Qiu et al., 2020), leukemia (Piccinini et al., 2005; Meier-Stephenson et al., 2017), lung cancer (Yang et al., 2006; Rengan et al., 2019), breast cancer (Srirangam et al., 2006; Soprano et al., 2016), glioblastoma (Pajonk et al., 2002; Rauschenbach et al., 2020), multiple myeloma (Ikezoe et al., 2004; Mendez-Lopez et al., 2019), melanoma (Jiang et al., 2007; Paskas et al., 2019), and ovarian cancer (Kumar et al., 2009; Perna et al., 2017).

HIV-PIs such as indinavir and saquinavir have shown antineoplastic potential in human tumours, such as hepatic, colon, lung and breast adenocarcinomas by blocking matrix metalloproteinases (MMPs) activity, which facilitate angiogenesis by degrading basement membranes, permitting endothelial cell invasion (Spugnini et al., 2006; Kumar et al., 2009; Toschi et al., 2011). In addition, these protease inhibitors impaired cellular proteasome by targeting its chymotrypsin activity which affects the rate of protein break down (Gaedicke et al., 2002; Chow et al., 2009; Toschi et al., 2011). Interestingly, proteasome inhibition by HIV-PIs prevented E6 induced p53 degradation in cervical cancer cells (Hampson et al., 2006). Through these mechanisms, HIV protease inhibitors increased the levels of growth-suppressive proteins in tumour cells, thus arresting tumour cell growth and/or stimulating apoptosis in tumour cells by inducing the expression of cell cycle and apoptosis regulators such as reactive oxygen species (ROS) and p21 (Gaedicke et al., 2002; Hampson et al., 2006; Chow et al., 2009; Toschi et al., 2011, Xiang et al., 2015). The common mechanisms of HIV protease inhibitors for preventing cervical cancer development and progression are summarised in Table 2. The table highlights cancer processes that are modulated by HIV protease inhibitors; these processes include cell invasion, apoptosis and cell cycle. To regulate cancer related processes, HIV protease inhibitors augment the expression of p53 (Park et al., 2021).

**TABLE 2:** The mechanisms of different HIV protease inhibitors in cervical cancer.

HIV protease inhibitor	Mode of action	References
Apoptosis inducers		
Nelfinavir	Induces apoptosis by stimulating the production of ROS	Xiang et al. (2015)
Cell cycle arrest inducers		
Nelfinavir	Induces cell cycle arrest at G1 phase by stimulating the production of ROS	Xiang et al. (2015)
Protect p53 from degradation		
Lopinavir	Inhibits E6-mediated proteasomal degradation of p53	Park et al. (2021)
Indinavir	Inhibits E6-mediated proteasomal degradation of p53	Hampson et al. (2006)
Ritonavir	Inhibits E6-mediated proteasomal degradation of p53 by reducing E6 and E7 protein levels	Barillari et al. (2012); Park et al. (2021)
Saquinavir	Inhibits E6-mediated proteasomal degradation of p53 by reducing E6 and E7 protein levels	Bandiera et al. (2016); Park et al. (2021)
Cell invasion inhibitors		
Ritonavir	Inhibits cell invasion by redcing the expression of matrix metalloproteinase (MMP)-2	Barillari et al. (2012); Park et al. (2021)
Saquinavir	Inhibits cell invasion by reducing matrix metalloproteinase(MMP)-2	Barillari et al. (2012); Park et al. (2021)

### HIV-PIs Restore p53 Expression in HPV-Associated Cervical Cancer

Since the pathogenicity of HPV-associated cancer involves function of viral E6 oncoprotein, HPV-associated neoplasms may be treated effectively by selectively blocking E6-dependent degradation of tumour suppressors and other cell homeostasis-related role players (Stuqui et al., 2016; Sharma and Munger, 2020; Gusho and Laimins, 2021). Previous studies have shown that there are inhibitors of E6/E6AP-mediated p53 degradation such as Pitx2 (Wei, 2005) and RITA (Zhao et al., 2010). These inhibitors induce the destruction of the E6/E6AP complex to restore the transcriptional function of p53, they do so by binding to E6 protein and blocking it to access E6AP and p53 thus preventing E6 mediated p53 proteasomal degradation (Wei, 2005; Zhao et al., 2010). As potential anticancer drugs, HIV protease inhibitors have also been shown to cause selective inhibition of the 26S proteasome (Piccinini et al., 2002; Lv et al., 2015). A foundation has been laid to demonstrate that some HIV-PIs can inhibit HPV-mediated degradation of p53 and induce apoptosis in HIV-associated cervical cancer cells (Hampson et al., 2006). Park et al. (2021) showed that a subset of HIV-1 protease inhibitors (nelfinavir, saquinavir, lopinavir and ritonavir) reduced the levels of HPV16 E6 and E7 oncoproteins in both CaSki and NIKS16 cells. The reduction of E6 and E7 oncoproteins correlated with increased levels of wild type p53 and HPV-positive cervical cancer cell death. Restored p53 resulted with enhanced apoptosis and accumulation of cells arrested in the G1 phase (Park et al., 2021).

However, different mechanisms on how HIV protease inhibitors restore the expression of p53 in HPV-associated cervical cancer remains unclear because studies in this field are limited. This has risen more questions, especially on the mechanisms used by HIV protease inhibitors to modulate p53 expression in cancer and the role players involved. Therefore, more studies are required both *in vitro* and *in vivo* to shed light on the clear mechanisms and role players involved in the restoration of p53 by HIV protease inhibitors. Previously, Hampson et al. (2006) attempted to answer these questions by examining whether the wild-type p53 that accumulates in cervical cancer cells after treatment with lopinavir is functional by examining the expression of the p53-transactivated gene, p21. p21 is an inhibitor of cyclin-dependent kinases, which are required to permit cell cycle progression. Surprisingly, there was no increase in the levels of p21 protein (Hampson et al., 2006). However, other studies showed that in some cell lines, E6 is able to downregulate p21, independently of p53 by inactivating p150^Sal2^, a p53-independent positive regulator of p21 transcription (Burkhart et al., 1999; Fan et al., 2005; Parroche et al., 2011).

Another study in accordance with Hampson et al. (2006) showed that HIV protease inhibitors caused evident depletion of E6 and E7, which correlated with increased p53 levels associated with anticancer properties in HPV-positive cancer cells (Park et al., 2021). Additionally, McFarlane et al. (2015) indicated that there are other ways to regulate p53 in HPV-associated cervical cells by targeting serine/arginine-rich splicing factors (SRSFs). The study showed that the depletion of SRSF2 resulted in a marked reduction in E6 and E7 transcript levels leading to induction of p53 expression, activity and stability.

### Molecular Mechanisms of p53-dependent Anticancer Activities Induced by HIV Protease Inhibitors

To promote cancer, HPV oncoprotein E6 through E6AP ubiquitinates p53 labelling it for degradation by the 26S proteasome (White et al., 2012; Yuan et al., 2012; Mesri et al., 2014). These effects remove the p53 and Rb-mediated G1 cell cycle checkpoint arrest (Lomazzi et al., 2002), thus promoting carcinogenesis. HIV protease inhibitors restore cell-cycle arrest and apoptosis, which are the most prominent outcomes of p53 activation (Qiu et al., 2020). In addition to their direct tumouricidal effects against cervical cancer cells, HIV-PIs have also been shown to curb the growth of lung cancer (Srirangam et al., 2011), breast cancer (Shim et al., 2012), colon cancer (Subeha and Telleria, 2020), and hepatic origin adenocarcinomas (Sun et al., 2012) by inhibiting the growth of new blood vessels that support the growth of tumours and their spread to other organs (Toschi et al., 2011).

**FIGURE 5:**
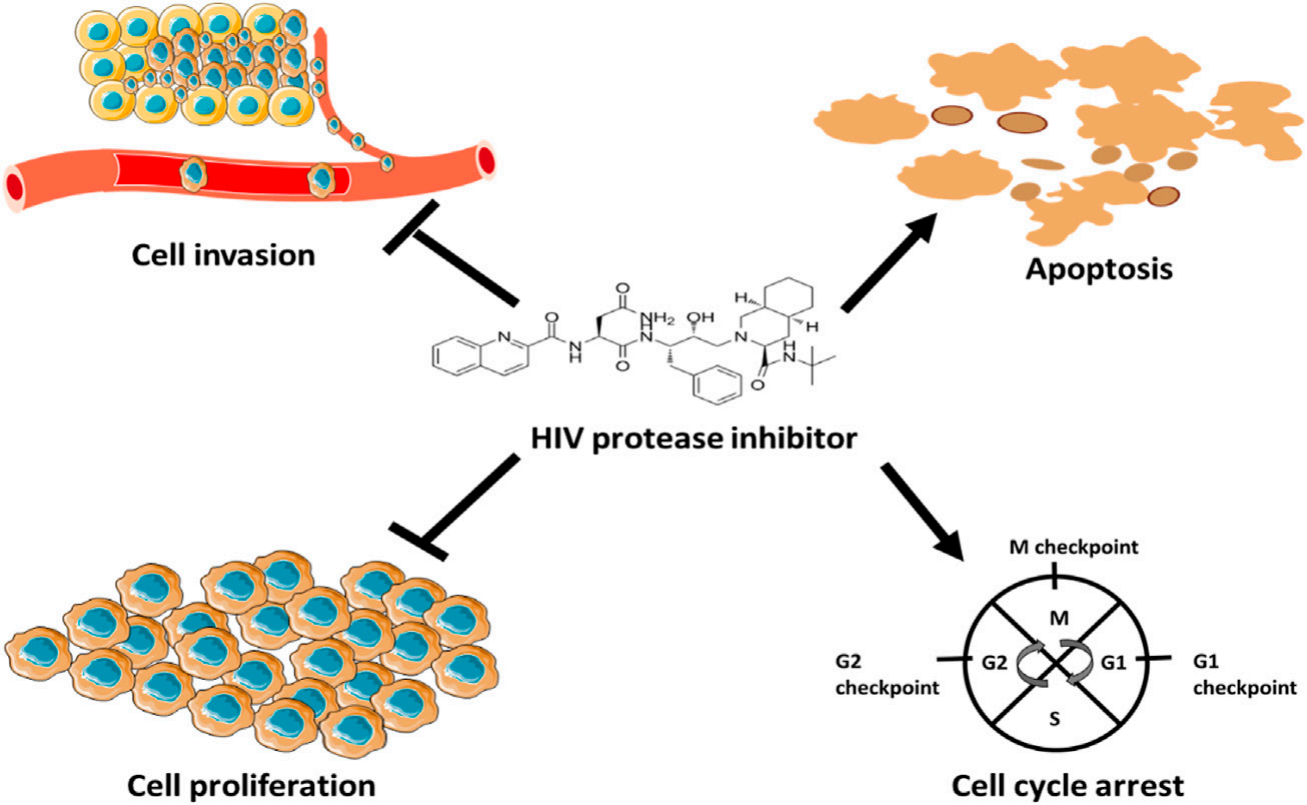
Anticancer mechanisms of HIV protease inhibitors. HIV protease inhibitors induce anticancer mechanisms by blocking MMPs activity and stimulating the production ROS. MMPs facilitate angiogenesis and ROS regulate cell cycle and trigger cytochrome *c* release to induce apoptosis.

Xiang et al. (2015) found that nelfinavir did not only promote apoptosis in HPV-positive cervical cancer cells, but also induced G1 cell cycle arrest. According to Xiang et al. (2015), nelfinavir induced apoptosis and cell cycle arrest by stimulating the production of ROS, which trigger cytochrome c release from mitochondria during apoptosis. Based on the discussion above, HIV-PIs have valuable therapeutic effects in haematological and solid cancers due to their inhibitory effects on tumour cell growth, invasion and angiogenesis and their ability to induce apoptosis and cell cycle arrest (Figure 5). Therefore, since the suppression of cancer cell growth can be caused either by the induction of apoptosis or cell cycle arrest mechanisms, the ability of HIV protease inhibitors to fine-tune p53 apoptosis and cell-cycle arrest activities should be explored further for therapeutic purposes.

### Other HPV-Mediated Deregulated p53-Related Mechanism During Carcinogenesis

Through its oncoproteins, HPV can evade the immune response of the host in different ways (Bonab et al., 2021). HPV oncoproteins also influence cell survival pathways, which target p53. HPV oncoproteins, E5 and E6, have been shown to regulate the activation and augmentation of epidermal growth factor receptor (EGFR), which is associated with poor prognosis in cervical cancer (Akerman et al., 2001; Iida et al., 2011; Ilahi and Bhatti, 2020). EGFR activates downstream oncogenesis-stimulating pathways such as PI3K and MAPK pathways (Zhou et al., 2015; Wee and Wang, 2017). Cancer cells rely on these pathways for differentiation, mitogenesis, survival and mobility (Wee and Wang 2017). p53-dependent anticancer mechanisms are counteracted by aberrant activation of PI3K and MAPK signalling pathways, because these signalling pathways are involved in promoting cell survival (Hanel et al., 2013; Martini et al., 2014; Guo et al., 2020). There is evidence indicating that cells harbouring high levels of the mutant protein p53 have elevated PI3K/Akt activity (Hanel et al., 2013); therefore targeting these pathways can be a promising strategy to halt the progression of HPV positive cancer cells. PI3K/Akt signalling pathways are activated by HPV infection accompanied by E6/E7 expression, which affects multiple cellular events that contribute to cancer development (Rodon et al., 2013; Manzo-Merino et al., 2014). Rodon et al. (2013) showed that HPV-associated laryngeal papillomas exhibited significant high activated levels of PI3K, which was accompanied by an increase in EGFR and subsequent activation of MAPK/ERK pathways. To promote cancer, HPV oncoproteins do not only affect survival pathways, they also targets other proteins whose defects cause cancer and other diseases (Bonab et al., 2021).

### HPV E6 Targets PDZ Proteins for Degradation

Contrary to low-risk HPVs, the cancer-causing high-risk HPVs’ E6s possess the PDZ-domain binding motif (PBM) at their C-termini (Banks et al., 2003; Subbaiah et al., 2011; Morgan et al., 2021), which mediate the interaction between HPV E6 and PDZ binding-domain containing proteins, such as NHERF-2 (Saidu et al., 2019), MAGI-1 (Araujo-Arcos et al., 2022), MAGI-2 (Thomas et al., 2002) and MAGI-3 (Ainsworth et al., 2008). The HPV E6 targets these PDZ domain containing proteins for degradation through the 26S proteasome (Massimi et al., 2008). PBM-mediated E6 interactions with PDZ-domain containing proteins play a role in both viral life cycles and the ability of the viruses to cause cell transformations and contribute to cancer development in transgenic mice (Banks et al., 2012; Yoshimatsu et al., 2017; Morgan et al., 2021). Additionally, the PBM also contains a phospho-acceptor site that has been demonstrated to negatively regulate the interaction of E6 with its PDZ domain-containing substrates (Boon and Banks, 2013). This phospho-acceptor site is also important for a feedback loop regulation between p53 and E6 (Vats et al., 2022). This finding suggests the complexity of the relation between p53 and HPV E6, and this is further complicated by E6 interaction with PDZ domain-containing proteins and the phosphorylation of E6.

Phosphorylated E6 shifts from interacting with PDZ domain-containing proteins to interact with proteins in the 14-3-3 family (Boon and Banks, 2013). In light of the knowledge that certain 14-3-3 isoforms are required for optimum p53 activity, this was particularly intriguing because there is evidence that 14-3-3σ regulates p53 subcellular distribution whereas 14-3-3ε and14-3-3γ are involved in stimulating the binding of p53 to p53 regulated promoters (Lee and Lozano, 2006; Rajagopalan et al., 2010; Falcicchio et al., 2020). A positive outcome of p53 and 14-3-3 protein interactions is also the protection of p53 from MDM2-mediated proteasomal degradation, which leads to an increase in p53 transcriptional activity and cell cycle arrest, thus restoring the tumour-suppressing properties of p53 (Yang et al., 2003; Rajagopalan et al., 2008; Falcicchio et al., 2020). To promote cancer, E6 target other isoforms of the 14-3-3 such as 14-3-3ζ which regulates multiple biological pathways that are involved in cancer progression (Boon and Banks, 2013). Overexpression of 14-3-3ζ has been detected in several human cancers including cervical cancer (Higareda-Almaraz et al., 2011), suggesting that it may be an oncogene. The complexity of the interaction between HPV E6 and other proteins emerged further when studies showed that HPV also regulates p53-associated lncRNAs (Mattick and Makunin, 2006; Sharma and Munger, 2020).

#### HPV Regulate p53-Associated lncRNAs

To date, only a limited number of lncRNAs have been found to be associated with cervical cancer (Zhu et al., 2017; Wu et al., 2018) and some have been linked to HPV oncoproteins, E6 (Sharma and Munger, 2018; Barr et al., 2019) or E7 (Sharma et al., 2015; He et al., 2019b). As a consequence of HPV infection, various lncRNAs are altered (Ma et al., 2017) and lncRNAs act as a bridge between HPV infection and downstream signalling pathways involved in cell death and cell survival (Cao et al., 2014; Jiang et al., 2015; de Carvalho Galvão and Coimbra, 2020). In some reports, HPV have been shown to modulate the expression of lncRNAs in cervical cancer, independent of the known targets of HR-HPV oncoproteins, p53/E6AP, the altered lncRNAs in cervical cancer include Fanconi anemia complementation group-2 (FANCI-2) (Liu et al., 2021), Family With Sequence Similarity 83 Member H antisense RNA 1 (FAM83H-AS1) (Barr et al., 2019) and Thymopoietin pseudogene 2 (TMPOP2) (He et al., 2019b). The expression of these lncRNAs are associated with increased cell proliferation suggesting a crucial role in cervical cancer progression (Barr et al., 2019; He et al., 2019b).

Since HPV elude immune response of the host in different ways, it is possible that it does so by altering the expression of p53-associated lncRNAs. Sharma and Munger (2020) showed that in HPV E6/E7-expressing-human foreskin keratinocytes (HFKs), DNA damage induced noncoding (DINO) lncRNA expression was lower than in control HFKs. The decrease in DINO expression is understandable because DINO is a p53 transcriptional target, amplifying p53-mediated signalling (Schmitt et al., 2016). Therefore, its depletion renders the cells more resistant to cell death caused by metabolic stress or chemotherapy drugs (Sharma and Munger, 2020). HPV E6/E7 also decreased the expression of maternally expressed gene 3 (MEG3) lncRNA (Harden et al., 2017). MEG3 acts as an activator of TP53 (Zhou et al., 2007) and its expression in cervical cancer cells inhibited proliferation, enhanced apoptosis, and reduced tumourigenesis (Zhang and Gao, 2019). Together these results shows the potential of lncRNAs as an option that can be considered to interrupt HPV mediated p53 degradation.

## Concluding Remarks

HPV infection is involved in the ongoing burden of cancer in low and middle-income countries. HPV encode proteins which promote cancer by degrading the tumour suppressor p53 and new anticancer drugs can be developed to target p53 restoration. Therefore, there is a need to identify new key role players to rescue p53 from HPV degradation. Some of these key role players is HIV protease inhibitors, which showed strong promise for therapeutic purposes. However, studies on the effect of HIV protease inhibitors in the restoration of p53 in HPV-associated cervical cancer are limited with inconsistent results. Therefore, more research is required in this field to bridge this gap. Additionally, lncRNAs, splicing factors, PDZ and 14-3-3 proteins hold potential to serve as alternatives that can be targeted to restore p53 expression in HPV-associated cancers.

## References

[B1] Abboodi F. Buckhaults P. Altomare D. Liu C. Hosseinipour M. Banister C. E. (2021). HPV-inactive Cell Populations Arise from HPV16-Transformed Human Keratinocytes after P53 Knockout. Virology 554, 9–16. 10.1016/j.virol.2020.12.005 33321328

[B191] Adler D. H. Kakinami L. Modisenyane T. Tshabangu N. Mohapi L. De Bruyn G. (2012). Increased Regression And Decreased Incidence Of Human Papillomavirus-Related Cervical Lesions Among HIV-Infected Women on HAART. AIDS 26(13), 1645–1652. 10.1097/qad.0b013e32835536a3 22555167 PMC3709565

[B2] Agalliu I. Gapstur S. Chen Z. Wang T. Anderson R. L. Teras L. (2016). Associations of Oral α-, β-, and γ-Human Papillomavirus Types with Risk of Incident Head and Neck Cancer. JAMA Oncol. 2 (5), 599–606. 10.1001/jamaoncol.2015.5504 26794505 PMC4956584

[B3] Ainsworth J. Thomas M. Banks L. Coutlee F. Matlashewski G. (2008). Comparison of P53 and the PDZ Domain Containing Protein MAGI-3 Regulation by the E6 Protein from High-Risk Human Papillomaviruses. Virol. J. 5 (1), 67–69. 10.1186/1743-422X-5-67 18518978 PMC2442060

[B4] Akerman G. S. Tolleson W. H. Brown K. L. Zyzak L. L. Mourateva E. Engin T. S. (2001). Human Papillomavirus Type 16 E6 and E7 Cooperate to Increase Epidermal Growth Factor Receptor (EGFR) mRNA Levels, Overcoming Mechanisms by Which Excessive EGFR Signaling Shortens the Life Span of normal Human Keratinocytes. Cancer Res. 61 (9), 3837–3843. 11325860

[B5] Akokuwebe M. E. Idemudia E. S. Lekulo A. M. Motlogeloa O. W. (2021). Determinants and Levels of Cervical Cancer Screening Uptake Among Women of Reproductive Age in South Africa: Evidence from South Africa Demographic and Health Survey Data, 2016. BMC Public Health 21 (1), 2013–2014. 10.1186/s12889-021-12020-z 34740352 PMC8571865

[B197] Anbarasan T. Bourdon J. C. (2019). The Emerging Landscape of p53 Isoforms In Physiology, Cancer And Degenerative Diseases. Int. J. Mol. Sci. 20(24), 6257. 10.3390/ijms20246257 PMC6941119 31835844

[B6] Araujo-Arcos L. E. Montaño S. Bello-Rios C. Garibay-Cerdenares O. L. Leyva-Vázquez M. A. Illades-Aguiar B. (2022). Molecular Insights into the Interaction of HPV-16 E6 Variants against MAGI-1 PDZ1 Domain. Sci. Rep. 12 (1), 1898–1914. 10.1038/s41598-022-05995-1 35115618 PMC8814009

[B7] Aubrey B. J. Kelly G. L. Janic A. Herold M. J. Strasser A. (2018). How Does P53 Induce Apoptosis and How Does This Relate to P53-Mediated Tumour Suppression? Cell Death Differ 25 (1), 104–113. 10.1038/cdd.2017.169 29149101 PMC5729529

[B8] Banister C. E. Liu C. Pirisi L. Creek K. E. Buckhaults P. J. (2017). Identification and Characterization of HPV-independent Cervical Cancers. Oncotarget 8 (8), 13375–13386. 10.18632/oncotarget.14533 28077784 PMC5355105

[B9] Banks L. Pim D. Thomas M. (2012). Human Tumour Viruses and the Deregulation of Cell Polarity in Cancer. Nat. Rev. Cancer 12 (12), 877–886. 10.1038/nrc3400 23175122

[B10] Banks L. Pim D. Thomas M. (2003). Viruses and the 26S Proteasome: Hacking into Destruction. Trends Biochemical Sciences 28 (8), 452–459. 10.1016/s0968-0004(03)00141-5 12932734

[B11] Barillari G. Iovane A. Bacigalupo I. Palladino C. Bellino S. Leone P. (2012). Ritonavir or Saquinavir Impairs the Invasion of Cervical Intraepithelial Neoplasia Cells via a Reduction of MMP Expression and Activity. Aids 26 (8), 909–919. 10.1097/qad.0b013e328351f7a5 22313963

[B12] Barillari G. Sgadari C. Toschi E. Monini P. Ensoli B. (2003). HIV Protease Inhibitors as New Treatment Options for Kaposi's Sarcoma. Drug Resist. updates 6 (4), 173–181. 10.1016/s1368-7646(03)00060-8 12962683

[B13] Barr J. A. Hayes K. E. Brownmiller T. Harold A. D. Jagannathan R. Lockman P. R. (2019). Long Non-coding RNA FAM83H-AS1 Is Regulated by Human Papillomavirus 16 E6 Independently of P53 in Cervical Cancer Cells. Sci. Rep. 9 (1), 3662–3711. 10.1038/s41598-019-40094-8 30842470 PMC6403315

[B14] Baussano I. Franceschi S. Gillio-Tos A. Carozzi F. Confortini M. Dalla Palma P. (2013). Difference in Overall and Age-specific Prevalence of High-Risk Human Papillomavirus Infection in Italy: Evidence from NTCC Trial. BMC Infect. Dis. 13 (1), 238–8. 10.1186/1471-2334-13-238 23706168 PMC3669053

[B185] Bandiera E. Todeschini P. Romani C. Zanotti L. Erba E. Colmegna B. (2016). The Hiv‐Protease Inhibitor Saquinavir Reduces Proliferation, Invasion And Clonogenicity In Cervical Cancer Cell Lines. Oncol. Lett. 12(4), 2493–2500. 10.3892/ol.2016.5008 27698818 PMC5038480

[B15] Becerril S. Corchado-Cobos R. García-Sancha N. Revelles L. Revilla D. Ugalde T. (2021). Viruses and Skin Cancer. Int. J. Mol. Sci. 22 (10), TP5399. 10.3390/ijms22105399 PMC8161099 34065594

[B16] Bernard X. Robinson P. Nominé Y. Masson M. Charbonnier S. Ramirez-Ramos J. R. (2011). Proteasomal Degradation of P53 by Human Papillomavirus E6 Oncoprotein Relies on the Structural Integrity of P53 Core Domain. PloS one 6 (10), e25981. 10.1371/journal.pone.0025981 22046250 PMC3203139

[B17] Bernstein W. B. Dennis P. A. (2008). Repositioning HIV Protease Inhibitors as Cancer Therapeutics. Curr. Opin. HIV AIDS 3 (6), 666–675. 10.1097/coh.0b013e328313915d 19373040 PMC2682221

[B187] Blitz S. Baxter J. Raboud J. Walmsley S. Rachlis A. Smaill F. (2013). Evaluation of HIV and Highly Active Antiretroviral Therapy On The Natural History Of Human Papillomavirus Infection And Cervical Cytopathologic Findings In HIV-Positive And High-Risk HIV-Negative Women. J. Infect. Dis. 208(3), 454–462. 10.1093/infdis/jit181 23624362

[B18] Boon S. S. Banks L. (2013). High-Risk Human Papillomavirus E6 Oncoproteins Interact with 14-3-3ζ in a PDZ Binding Motif-dependent Manner. J. Virol. 87 (3), 1586–1595. 10.1128/jvi.02074-12 23175360 PMC3554170

[B19] Botha M. H. Dochez C. (2012). Introducing Human Papillomavirus Vaccines into the Health System in South Africa. Vaccine 30, C28–C34. 10.1016/j.vaccine.2012.03.032 22939017

[B20] Brancaccio R. N. Robitaille A. Dutta S. Cuenin C. Santare D. Skenders G. (2018). Generation of a Novel Next-Generation Sequencing-Based Method for the Isolation of New Human Papillomavirus Types. Virology 520, 1–10. 10.1016/j.virol.2018.04.017 29747121 PMC9280450

[B21] Bray F. Ferlay J. Soerjomataram I. Siegel R. L. Torre L. A. Jemal A. (2018). Global Cancer Statistics 2018: GLOBOCAN Estimates of Incidence and Mortality Worldwide for 36 Cancers in 185 Countries. CA: a Cancer J. clinicians 68 (6), 394–424. 10.3322/caac.21492 30207593

[B74] Broz D. K. Mello S. S. Bieging K. T. Jiang D. Dusek R. L. Brady C. A. (2013). Global Genomic Profiling Reveals an Extensive P53-Regulated Autophagy Program Contributing to Key P53 Responses. Genes Dev. 27 (9), 1016–1031. 10.1101/gad.212282.112 23651856 PMC3656320

[B22] Burkhart B. A. Alcorta D. A. Chiao C. Isaacs J. S. Barrett J. C. (1999). Two Posttranscriptional Pathways that Regulate p21Cip1/Waf1/Sdi1Are Identified by HPV16-E6 Interaction and Correlate with Life Span and Cellular Senescence. Exp. Cel. Res. 247 (1), 168–175. 10.1006/excr.1998.4345 10047459

[B23] Cameron J. E. Rositch A. F. Vielot N. A. Mugo N. R. Kwatampora J. K. L. Waweru W. (2018). Epstein-Barr Virus, High-Risk Human Papillomavirus and Abnormal Cervical Cytology in a Prospective Cohort of African Female Sex Workers. Sex. Trans. Dis. 45 (10), 666–672. 10.1097/olq.0000000000000857 PMC6482813 29664764

[B24] Cao S. Liu W. Li F. Zhao W. Qin C. (2014). Decreased Expression of lncRNA GAS5 Predicts a Poor Prognosis in Cervical Cancer. Int. J. Clin. Exp. Pathol. 7 (10), 6776–6783. 25400758 PMC4230116

[B25] Chae Y. K. Anker J. F. Carneiro B. A. Chandra S. Kaplan J. Kalyan A. (2016). Genomic Landscape of DNA Repair Genes in Cancer. Oncotarget 7 (17), 23312–23321. 10.18632/oncotarget.8196 27004405 PMC5029628

[B26] Cheng L. Wang Y. Du J. (2020). Human Papillomavirus Vaccines: An Updated Review. Vaccines 8 (3), 391. 10.3390/vaccines8030391 PMC7565290 32708759

[B27] Chesson H. W. Dunne E. F. Hariri S. Markowitz L. E. (2014). The Estimated Lifetime Probability of Acquiring Human Papillomavirus in the United States. Sex. Transm. Dis. 41 (11), 660–664. 10.1097/olq.0000000000000193 25299412 PMC6745688

[B28] Chipuk J. E. Green D. R. (2005). Do inducers of Apoptosis Trigger Caspase-independent Cell Death? Nat. Rev. Mol. Cel Biol 6 (3), 268–275. 10.1038/nrm1573 15714200

[B29] Chow W. A. Jiang C. Guan M. (2009). Anti-HIV Drugs for Cancer Therapeutics: Back to the Future? Lancet Oncol. 10 (1), 61–71. 10.1016/s1470-2045(08)70334-6 19111246

[B30] Cobzeanu B. M. Popescu E. Danciu M. Paşca A. S. Palade O. D. Vonica S. P. (2019). Correlations between HPV, P53 and P16 in Malignancies Involving the Retromolar Trigone-Oropharynx junction. Rom. J. Morphol. Embryol. 60, 853–859. 31912096

[B31] Cornall A. M. Roberts J. M. Garland S. M. Hillman R. J. Grulich A. E. Tabrizi S. N. (2013). Anal and Perianal Squamous Carcinomas and High-Grade Intraepithelial Lesions Exclusively Associated with "Low-Risk" HPV Genotypes 6 and 11. Int. J. Cancer 133 (9), 2253–2258. 10.1002/ijc.28228 23616200

[B32] Crighton D. Wilkinson S. O'Prey J. Syed N. Smith P. Harrison P. R. (2006). DRAM, a P53-Induced Modulator of Autophagy, Is Critical for Apoptosis. Cell 126 (1), 121–134. 10.1016/j.cell.2006.05.034 16839881

[B33] Dalton M. Holzman E. Erwin E. Michelen S. Rositch A. F. Kumar S. (2019). Patient Navigation Services for Cancer Care in Low-And Middle-Income Countries: a Scoping Review. PLoS One 14 (10), e0223537. 10.1371/journal.pone.0223537 31622363 PMC6797131

[B34] de Carvalho Galvão M. L. T. Coimbra E. C. (2020). Long Noncoding RNAs (lncRNAs) in Cervical Carcinogenesis: New Molecular Targets, Current Prospects. Crit. Rev. Oncology/Hematology 156, 103111. 10.1016/j.critrevonc.2020.103111 33080526

[B35] de Martel C. Plummer M. Vignat J. Franceschi S. (2017). Worldwide burden of Cancer Attributable to HPV by Site, Country and HPV Type. Int. J. Cancer 141 (4), 664–670. 10.1002/ijc.30716 28369882 PMC5520228

[B36] de Sanjosé S. Brotons M. Pavón M. A. (2018). The Natural History of Human Papillomavirus Infection. Best Pract. Res. Clin. Obstet. Gynaecol. 47, 2–13. 10.1016/j.bpobgyn.2017.08.015 28964706

[B37] De Villiers E.-M. Fauquet C. Broker T. R. Bernard H.-U. zur Hausen H. (2004). Classification of Papillomaviruses. Virology 324, 17–27. 10.1016/j.virol.2004.03.033 15183049

[B38] Denk C. Butz K. Schneider A. Dürst M. Hoppe-Seyler F. (2001). p53 Mutations Are Rare Events in Recurrent Cervical Cancer. J. Mol. Med. (Berl) 79 (5), 283–288. 10.1007/s001090100191 11485021

[B39] Eischen C. M. (2016). Genome Stability Requires P53. Cold Spring Harb Perspect. Med. 6 (6), a026096. 10.1101/cshperspect.a026096 27252396 PMC4888814

[B40] Falcicchio M. Ward J. A. Macip S. Doveston R. G. (2020). Regulation of P53 by the 14-3-3 Protein Interaction Network: New Opportunities for Drug Discovery in Cancer. Cell Death Discov 6 (1), 126–221. 10.1038/s41420-020-00362-3 33298896 PMC7669891

[B41] Fan X. Liu Y. Chen J. J. (2005). Down-regulation of P21 Contributes to Apoptosis Induced by HPV E6 in Human Mammary Epithelial Cells. Apoptosis 10 (1), 63–73. 10.1007/s10495-005-6062-y 15711923

[B42] Feng X. Lu H. Wei Y. Guan M. Wang J. Liu C. (2021). Prognostic Impact of Hepatitis B Virus Infection in Patients with Primary Cervical Cancer. Cancer Med. 10 (23), 8310–8319. 10.1002/cam4.4358 34672431 PMC8633261

[B43] Feroz W. Sheikh A. M. A. (2020). Exploring the Multiple Roles of Guardian of the Genome: P53. Egypt. J. Med. Hum. Genet. 21 (1), 1–23. 10.1186/s43042-020-00089-x

[B44] Gaedicke S. Firat-Geier E. Constantiniu O. Lucchiari-Hartz M. Freudenberg M. Galanos C. (2002). Antitumor Effect of the Human Immunodeficiency Virus Protease Inhibitor Ritonavir: Induction of Tumor-Cell Apoptosis Associated with Perturbation of Proteasomal Proteolysis. Cancer Res. 62 (23), 6901–6908. 12460905

[B45] Gantt S. Carlsson J. Ikoma M. Gachelet E. Gray M. Geballe A. P. (2011). The HIV Protease Inhibitor Nelfinavir Inhibits Kaposi's Sarcoma-Associated Herpesvirus Replication *In Vitro* . Antimicrob. Agents Chemother. 55 (6), 2696–2703. 10.1128/aac.01295-10 21402841 PMC3101462

[B46] Gao W. Shen Z. Shang L. Wang X. (2011). Upregulation of Human Autophagy-Initiation Kinase ULK1 by Tumor Suppressor P53 Contributes to DNA-Damage-Induced Cell Death. Cel Death Differ 18 (10), 1598–1607. 10.1038/cdd.2011.33 PMC3172118 21475306

[B47] Garima S. Pandey S. Pandey L. K. Saxena A. K. Patel N. (2016). The Role of P53 Gene in Cervical Carcinogenesis. J. Obstet. Gynaecol. India 66 (1), 383–388. 10.1007/s13224-015-0754-1 27651634 PMC5016398

[B48] Gratton R. Tricarico P. M. Guimaraes R. L. Celsi F. Crovella S. (2018). Lopinavir/Ritonavir Treatment Induces Oxidative Stress and Caspaseindependent Apoptosis in Human Glioblastoma U-87 MG Cell Line. Chr 16 (2), 106–112. 10.2174/1570162x16666180528100922 29804534

[B49] Guan P. Howell-Jones R. Li N. Bruni L. de Sanjosé S. Franceschi S. (2012). Human Papillomavirus Types in 115,789 HPV-Positive Women: A Meta-Analysis from Cervical Infection to Cancer. Int. J. Cancer 131 (10), 2349–2359. 10.1002/ijc.27485 22323075

[B50] Guo Y. J. Pan W. W. Liu S. B. Shen Z. F. Xu Y. Hu L. L. (2020). ERK/MAPK Signalling Pathway and Tumorigenesis. Exp. Ther. Med. 19 (3), 1997–2007. 10.3892/etm.2020.8454 32104259 PMC7027163

[B51] Gusho E. Laimins L. (2021). Human Papillomaviruses Target the DNA Damage Repair and Innate Immune Response Pathways to Allow for Persistent Infection. Viruses 13 (7), 1390. 10.3390/v13071390 34372596 PMC8310235

[B52] Hafner A. Bulyk M. L. Jambhekar A. Lahav G. (2019). The Multiple Mechanisms that Regulate P53 Activity and Cell Fate. Nat. Rev. Mol. Cel Biol 20 (4), 199–210. 10.1038/s41580-019-0110-x 30824861

[B53] Hampson L. Kitchener H. C. Hampson I. N. (2006). Specific HIV Protease Inhibitors Inhibit the Ability of HPV16 E6 to Degrade P53 and Selectively Kill E6-dependent Cervical Carcinoma Cells *In Vitro* . Antivir. Ther. 11 (6), 813–826. 10.1177/135965350601100607 17310826

[B54] Han N. Yuan F. Xian P. Liu N. Liu J. Zhang H. (2019). GADD45a Mediated Cell Cycle Inhibition Is Regulated by P53 in Bladder Cancer. Ott 12, 7591–7599. 10.2147/ott.s222223 PMC6754676 31571910

[B55] Hanel W. Marchenko N. Xu S. Xiaofeng Yu S. Weng W. Moll U. (2013). Two Hot Spot Mutant P53 Mouse Models Display Differential Gain of Function in Tumorigenesis. Cel Death Differ 20 (7), 898–909. 10.1038/cdd.2013.17 PMC3679454 23538418

[B56] Harden M. E. Prasad N. Griffiths A. Munger K. (2017). Modulation of microRNA-mRNA Target Pairs by Human Papillomavirus 16 Oncoproteins. MBio 8 (1), e02170–16. 10.1128/mBio.02170-16 28049151 PMC5210503

[B57] He F. Borcherds W. Song T. Wei X. Das M. Chen L. (2019a). Interaction between P53 N Terminus and Core Domain Regulates Specific and Nonspecific DNA Binding. Proc. Natl. Acad. Sci. U.S.A. 116 (18), 8859–8868. 10.1073/pnas.1903077116 30988205 PMC6500136

[B58] He H. Liu X. Liu Y. Zhang M. Lai Y. Hao Y. (2019b). Human Papillomavirus E6/E7 and Long Noncoding RNA TMPOP2 Mutually Upregulated Gene Expression in Cervical Cancer Cells. J. Virol. 93 (8), e01808–18. 10.1128/JVI.01808-18 30728257 PMC6450114

[B189] Heard I. Potard V. Costagliola D. (2006). Limited Impact of Immunosuppression and Heart on the Incidence of Cervical Squamous Intraepithelial Lesions in HIV-Positive Women. Antiviral Therapy 11(8), 1091–1096. 10.1177/135965350601100816 17302379

[B190] Heard I. Schmitz V. Costagliola D. Orth G. Kazatchkine M. D. (1998). Early Regression Of Cervical Lesions in HIV-Seropositive Women Receiving Highly Active Antiretroviral Therapy. Aids 12(12), 1459–1464. 10.1097/00002030-199812000-00007 9727566

[B59] Hengstermann A. Linares L. K. Ciechanover A. Whitaker N. J. Scheffner M. (2001). Complete Switch from Mdm2 to Human Papillomavirus E6-Mediated Degradation of P53 in Cervical Cancer Cells. Proc. Natl. Acad. Sci. U.S.A. 98 (3), 1218–1223. 10.1073/pnas.98.3.1218 11158620 PMC14735

[B60] Hernández-Ramírez R. U. Shiels M. S. Dubrow R. Engels E. A. (2017). Cancer Risk in HIV-Infected People in the USA from 1996 to 2012: a Population-Based, Registry-Linkage Study. Lancet HIV 4 (11), e495–e504. 10.1016/S2352-3018(17)30125-X 28803888 PMC5669995

[B61] Higareda-Almaraz J. C. Enríquez-Gasca Mdel. R. Hernández-Ortiz M. Resendis-Antonio O. Encarnación-Guevara S. (2011). Proteomic Patterns of Cervical Cancer Cell Lines, a Network Perspective. BMC Syst. Biol. 5 (1), 96–16. 10.1186/1752-0509-5-96 21696634 PMC3152905

[B62] Hoppe-Seyler K. Bossler F. Braun J. A. Herrmann A. L. Hoppe-Seyler F. (2018). The HPV E6/E7 Oncogenes: Key Factors for Viral Carcinogenesis and Therapeutic Targets. Trends Microbiology 26 (2), 158–168. 10.1016/j.tim.2017.07.007 28823569

[B63] Iida K. Nakayama K. Rahman M. T. Rahman M. Ishikawa M. Katagiri A. (2011). EGFR Gene Amplification Is Related to Adverse Clinical Outcomes in Cervical Squamous Cell Carcinoma, Making the EGFR Pathway a Novel Therapeutic Target. Br. J. Cancer 105 (3), 420–427. 10.1038/bjc.2011.222 21730982 PMC3172895

[B64] Ikezoe T. Saito T. Bandobashi K. Yang Y. Koeffler H. P. Taguchi H. (2004). HIV-1 Protease Inhibitor Induces Growth Arrest and Apoptosis of Human Multiple Myeloma Cells via Inactivation of Signal Transducer and Activator of Transcription 3 and Extracellular Signal-Regulated Kinase 1/2. Mol. Cancer Ther. 3 (4), 473–479. 10.1016/s1359-6349(04)80382-3 15078991

[B65] Ilahi N. E. Bhatti A. (2020). Impact of HPV E5 on Viral Life Cycle via EGFR Signaling. Microb. Pathogenesis 139, 103923. 10.1016/j.micpath.2019.103923 31836496

[B66] Jalil A. T. Karevskiy A. (2020). The Cervical Cancer (CC) Epidemiology and Human Papillomavirus (HPV) in the Middle East. Int. J. Environ. Eng. Educ. 2 (2), 7–12. 10.55151/ijeedu.v2i2.29

[B67] Jiang P. Yue Y. (2014). Human Papillomavirus Oncoproteins and Apoptosis (Review). Exp. Ther. Med. 7 (1), 3–7. 10.3892/etm.2013.1374 24348754 PMC3860870

[B68] Jiang S. Wang H. L. Yang J. (2015). Low Expression of Long Non-coding RNA LET Inhibits Carcinogenesis of Cervical Cancer. Int. J. Clin. Exp. Pathol. 8 (1), 806–811. 25755778 PMC4348863

[B69] Jiang W. Mikochik P. J. Ra J. H. Lei H. Flaherty K. T. Winkler J. D. (2007). HIV Protease Inhibitor Nelfinavir Inhibits Growth of Human Melanoma Cells by Induction of Cell Cycle Arrest. Cancer Res. 67 (3), 1221–1227. 10.1158/0008-5472.can-06-3377 17283158

[B70] Joruiz S. M. Bourdon J.-C. (2016). p53 Isoforms: Key Regulators of the Cell Fate Decision. Cold Spring Harb Perspect. Med. 6 (8), a026039. 10.1101/cshperspect.a026039 26801896 PMC4968168

[B71] Kabsch K. Alonso A. (2002). The Human Papillomavirus Type 16 E5 Protein Impairs TRAIL- and FasL-Mediated Apoptosis in HaCaT Cells by Different Mechanisms. J. Virol. 76 (23), 12162–12172. 10.1128/jvi.76.23.12162-12172.2002 12414956 PMC136856

[B72] Kariya R. Taura M. Suzu S. Kai H. Katano H. Okada S. (2014). HIV Protease Inhibitor Lopinavir Induces Apoptosis of Primary Effusion Lymphoma Cells via Suppression of NF-Κb Pathway. Cancer Lett. 342 (1), 52–59. 10.1016/j.canlet.2013.08.045 24012878

[B73] Kato S. Han S.-Y. Liu W. Otsuka K. Shibata H. Kanamaru R. (2003). Understanding the Function-Structure and Function-Mutation Relationships of P53 Tumor Suppressor Protein by High-Resolution Missense Mutation Analysis. Proc. Natl. Acad. Sci. U.S.A. 100 (14), 8424–8429. 10.1073/pnas.1431692100 12826609 PMC166245

[B188] Kelly H. Weiss H. A. Benavente Y. de Sanjose S. Mayaud P. Qiao Y. L. (2018). Association of Antiretroviral Therapy With High-Risk Human Papillomavirus, Cervical Intraepithelial Neoplasia, And Invasive Cervical Cancer In Women Living With HIV: A Systematic Review And Meta-Analysis. The Lancet HIV 5(1), e45–e58. 10.1016/s2352-3018(17)30149-2 29107561 PMC5757426

[B193] Kelly H. A. Sawadogo B. Chikandiwa A. Segondy M. Gilham C. Lompo O. (2017). Epidemiology of High-Risk Human Papillomavirus And Cervical Lesions in African Women Living With HIV/AIDS: Effect Of Anti-Retroviral Therapy. Aids 31(2), 273–285. 10.1097/qad.0000000000001301 27755107

[B75] Khoury M. P. Bourdon J.-C. (2011). p53 Isoforms: an Intracellular Microprocessor? Genes & cancer 2 (4), 453–465. 10.1177/1947601911408893 21779513 PMC3135639

[B76] Kim S. An S. S. A. (2016). Role of P53 Isoforms and Aggregations in Cancer. Medicine 95 (26), e3993. 10.1097/md.0000000000003993 27368003 PMC4937917

[B77] Kumar S. Bryant C. S. Chamala S. Qazi A. Seward S. Pal J. (2009). Ritonavir Blocks AKT Signaling, Activates Apoptosis and Inhibits Migration and Invasion in Ovarian Cancer Cells. Mol. Cancer 8 (1), 26–12. 10.1186/1476-4598-8-26 19386116 PMC2691728

[B78] Kushchayeva Y. Jensen K. Recupero A. Costello J. Patel A. Klubo-Gwiezdzinska J. (2014). The HIV Protease Inhibitor Nelfinavir Down-Regulates RET Signaling and Induces Apoptosis in Medullary Thyroid Cancer Cells. J. Clin. Endocrinol. Metab. 99 (5), E734–E745. 10.1210/jc.2013-3369 24483157

[B79] Lee M.-H. Lozano G. (2006). Regulation of the P53-MDM2 Pathway by 14-3-3 σ and Other Proteins. Semin. Cancer Biol. 16 (3), 225–234. 10.1016/j.semcancer.2006.03.009 16697215

[B80] Li L. Deng B. Xing G. Teng Y. Tian C. Cheng X. (2007). PACT Is a Negative Regulator of P53 and Essential for Cell Growth and Embryonic Development. Proc. Natl. Acad. Sci. U.S.A. 104 (19), 7951–7956. 10.1073/pnas.0701916104 17470788 PMC1876553

[B81] Li N. Franceschi S. Howell-Jones R. Snijders P. J. F. Clifford G. M. (2011). Human Papillomavirus Type Distribution in 30,848 Invasive Cervical Cancers Worldwide: Variation by Geographical Region, Histological Type and Year of Publication. Int. J. Cancer 128 (4), 927–935. 10.1002/ijc.25396 20473886

[B82] Li S. Hong X. Wei Z. Xie M. Li W. Liu G. (2019). Ubiquitination of the HPV Oncoprotein E6 Is Critical for E6/E6AP-Mediated P53 Degradation. Front. Microbiol. 10, 2483. 10.3389/fmicb.2019.02483 31749782 PMC6842930

[B83] Lin H. H. Zhang Q. R. Kong X. Zhang L. Zhang Y. Tang Y. (2021). Machine Learning Prediction of Antiviral-HPV Protein Interactions for Anti-HPV Pharmacotherapy. Sci. Rep. 11 (1), 24367–24368. 10.1038/s41598-021-03000-9 34934067 PMC8692573

[B84] List J. M. O'Connor J. M. (2020). How Should Low- and Middle-Income Countries Motivate Equity in Cancer Prevention and Control? AMA J. Ethics 22 (2), E147–E155. 10.1001/amajethics.2020.147 32048585

[B85] Liu H. Xu J. Yang Y. Wang X. Wu E. Majerciak V. (2021). Oncogenic HPV Promotes the Expression of the Long Noncoding RNA Lnc-FANCI-2 through E7 and YY1. Proc. Natl. Acad. Sci. 118 (3), e2014195118. 10.1073/pnas.2014195118 33436409 PMC7826414

[B86] Lomazzi M. Moroni M. C. Jensen M. R. Frittoli E. Helin K. (2002). Suppression of the P53- or pRB-Mediated G1 Checkpoint Is Required for E2F-Induced S-phase Entry. Nat. Genet. 31 (2), 190–194. 10.1038/ng891 11992123

[B87] Lv Z. Chu Y. Wang Y. (2015). HIV Protease Inhibitors: a Review of Molecular Selectivity and Toxicity. HIV AIDS (Auckl) 7, 95–104. 10.2147/HIV.S79956 25897264 PMC4396582

[B88] Ma X. Sheng S. Wu J. Jiang Y. Gao X. Cen X. (2017). LncRNAs as an Intermediate in HPV16 Promoting Myeloid-Derived Suppressor Cell Recruitment of Head and Neck Squamous Cell Carcinoma. Oncotarget 8 (26), 42061–42075. 10.18632/oncotarget.14939 28159935 PMC5522049

[B89] Maksimovic-Ivanic D. Fagone P. McCubrey J. Bendtzen K. Mijatovic S. Nicoletti F. (2017). HIV-protease Inhibitors for the Treatment of Cancer: Repositioning HIV Protease Inhibitors while Developing More Potent NO-Hybridized Derivatives? Int. J. Cancer 140 (8), 1713–1726. 10.1002/ijc.30529 27870005

[B90] Manzo-Merino J. Contreras-Paredes A. Vázquez-Ulloa E. Rocha-Zavaleta L. Fuentes-Gonzalez A. M. Lizano M. (2014). The Role of Signaling Pathways in Cervical Cancer and Molecular Therapeutic Targets. Arch. Med. Res. 45 (7), 525–539. 10.1016/j.arcmed.2014.10.008 25450584

[B91] Marcel V. Hainaut P. (2009). p53 Isoforms - A Conspiracy to Kidnap P53 Tumor Suppressor Activity? Cell. Mol. Life Sci. 66 (3), 391–406. 10.1007/s00018-008-8336-3 18854945 PMC11131440

[B92] Marima R. Hull R. Dlamini Z. Penny C. (2020). The Dual Protease Inhibitor Lopinavir/ritonavir (LPV/r) Exerts Genotoxic Stress on Lung Cells. Biomed. Pharmacother. 132, 110829. 10.1016/j.biopha.2020.110829 33059259

[B93] Martinez-Zapien D. Ruiz F. X. Poirson J. Mitschler A. Ramirez J. Forster A. (2016). Structure of the E6/E6AP/p53 Complex Required for HPV-Mediated Degradation of P53. Nature 529 (7587), 541–545. 10.1038/nature16481 26789255 PMC4853763

[B94] Martini M. De Santis M. C. Braccini L. Gulluni F. Hirsch E. (2014). PI3K/AKT Signaling Pathway and Cancer: an Updated Review. Ann. Med. 46 (6), 372–383. 10.3109/07853890.2014.912836 24897931

[B95] Massimi P. Shai A. Lambert P. Banks L. (2008). HPV E6 Degradation of P53 and PDZ Containing Substrates in an E6AP Null Background. Oncogene 27 (12), 1800–1804. 10.1038/sj.onc.1210810 17934525

[B96] Mattick J. S. Makunin I. V. (2006). Non-coding RNA. Hum. Mol. Genet. 15 (Suppl. l_1), R17–R29. 10.1093/hmg/ddl046 16651366

[B97] McFarlane M. MacDonald A. I. Stevenson A. Graham S. V. (2015). Human Papillomavirus 16 Oncoprotein Expression Is Controlled by the Cellular Splicing Factor SRSF2 (SC35). J. Virol. 89 (10), 5276–5287. 10.1128/jvi.03434-14 25717103 PMC4442513

[B195] McKenzie N. D. Kobetz E. N. Hnatyszyn J. Twiggs L. B. Lucci J. A. III (2010). Women with HIV are More Commonly Infected With Non-16 and-18 High-Risk HPV Types. Gynecol. Oncol. 116(3), 572–577. 10.1016/j.ygyno.2009.10.058 19906410

[B98] McLaughlin-Drubin M. E. Münger K. (2009). The Human Papillomavirus E7 Oncoprotein. Virology 384 (2), 335–344. 10.1016/j.virol.2008.10.006 19007963 PMC2661820

[B99] Mehta S. Campbell H. Drummond C. J. Li K. Murray K. Slatter T. (2021). Adaptive Homeostasis and the P53 Isoform Network. EMBO Rep. 22 (12), e53085. 10.15252/embr.202153085 34779563 PMC8647153

[B100] Meier-Stephenson V. Riemer J. Narendran A. (2017). The HIV Protease Inhibitor, Nelfinavir, as a Novel Therapeutic Approach for the Treatment of Refractory Pediatric Leukemia. Ott Vol. 10, 2581–2593. 10.2147/ott.s136484 PMC5440076 28553123

[B101] Mendez-Lopez M. Sutter T. Driessen C. Besse L. (2019). HIV Protease Inhibitors for the Treatment of Multiple Myeloma. Clin. Adv. Hematol. Oncol. 17, 615–623. 31851164

[B102] Mesri E. A. Feitelson M. A. Munger K. (2014). Human Viral Oncogenesis: a Cancer Hallmarks Analysis. Cell host & microbe 15 (3), 266–282. 10.1016/j.chom.2014.02.011 24629334 PMC3992243

[B103] Mirbahari S. Sadeghi M. (2018). The Prevalence of Genus Alpha Human Papillomavirus in Women with Uterine Cervical Infection And/or Inflammation in Western Iran. Mater. Sociomed 30 (2), 113. 10.5455/msm.2018.30.113-117 30061800 PMC6029900

[B104] Monini P. Sgadari C. Barillari G. Ensoli B. (2003). HIV Protease Inhibitors: Antiretroviral Agents with Anti-inflammatory, Anti-angiogenic and Anti-tumour Activity. J. Antimicrob. Chemother. 51 (2), 207–211. 10.1093/jac/dkg086 12562682

[B105] Morgan E. L. Scarth J. A. Patterson M. R. Wasson C. W. Hemingway G. C. Barba-Moreno D. (2021). E6-mediated Activation of JNK Drives EGFR Signalling to Promote Proliferation and Viral Oncoprotein Expression in Cervical Cancer. Cel Death Differ 28 (5), 1669–1687. 10.1038/s41418-020-00693-9 PMC8166842 33303976

[B106] Murray-Zmijewski F. Slee E. A. Lu X. (2008). A Complex Barcode Underlies the Heterogeneous Response of P53 to Stress. Nat. Rev. Mol. Cel Biol 9 (9), 702–712. 10.1038/nrm2451 18719709

[B107] Nagasaka K. Kawana K. Osuga Y. Fujii T. (2013). PDZ Domains and Viral Infection: Versatile Potentials of HPV-PDZ Interactions in Relation to Malignancy. Biomed. Research International 2013, 1–9. 10.1155/2013/369712 PMC3777178 24093094

[B108] Pajonk F. Himmelsbach J. Riess K. Sommer A. McBride W. H. (2002). The Human Immunodeficiency Virus (HIV)-1 Protease Inhibitor Saquinavir Inhibits Proteasome Function and Causes Apoptosis and Radiosensitization in Non-HIV-associated Human Cancer Cells. Cancer Res. 62 (18), 5230–5235. 12234989

[B109] Pandey P. Khan F. Farhan M. Jafri A. (2021). Elucidation of Rutin's Role in Inducing Caspase-dependent Apoptosis via HPV-E6 and E7 Down-Regulation in Cervical Cancer HeLa Cells. Biosci. Rep. 41 (6), BSR20210670. 10.1042/BSR20210670 34109976 PMC8220446

[B110] Park S. Auyeung A. Lee D. L. Lambert P. F. Carchman E. H. Sherer N. M. (2021). HIV-1 Protease Inhibitors Slow HPV16-Driven Cell Proliferation through Targeted Depletion of Viral E6 and E7 Oncoproteins. Cancers 13 (5), 949. 10.3390/cancers13050949 33668328 PMC7956332

[B111] Parrales A. Iwakuma T. (2015). Targeting Oncogenic Mutant P53 for Cancer Therapy. Front. Oncol. 5, 288. 10.3389/fonc.2015.00288 26732534 PMC4685147

[B112] Parroche P. Touka M. Mansour M. Bouvard V. Thépot A. Accardi R. (2011). Human Papillomavirus Type 16 E6 Inhibits p21WAF1 Transcription Independently of P53 by Inactivating p150Sal2. Virology 417 (2), 443–448. 10.1016/j.virol.2011.05.016 21791360

[B113] Paskas S. Mazzon E. Basile M. S. Cavalli E. Al-Abed Y. He M. (2019). Lopinavir-NO, a Nitric Oxide-Releasing HIV Protease Inhibitor, Suppresses the Growth of Melanoma Cells *In Vitro* and *In Vivo* . Invest. New Drugs 37 (5), 1014–1028. 10.1007/s10637-019-00733-3 30706336

[B114] Patton L. Ramirez-Amador V. Anaya-Saavedra G. Nittayananta W. Carrozzo M. Ranganathan K. (2013). Urban Legends Series: Oral Manifestations of HIV Infection. Oral Dis. 19 (6), 533–550. 10.1111/odi.12103 23517181

[B115] Perna A. Lucariello A. Sellitto C. Agliata I. Carleo M. A. Sangiovanni V. (2017). Different Cell Cycle Modulation in SKOV-3 Ovarian Cancer Cell Line by Anti-HIV Drugs. Oncol. Res. 25 (9), 1617–1624. 10.3727/096504017x14905635363102 28390117 PMC7841068

[B116] Piccinini M. Rinaudo M. T. Anselmino A. Buccinnà B. Ramondetti C. Dematteis A. (2005). The HIV Protease Inhibitors Nelfinavir and Saquinavir, but Not a Variety of HIV Reverse Transcriptase Inhibitors, Adversely Affect Human Proteasome Function. Antivir. Ther. 10 (2), 215–223. 10.1177/135965350501000203 15865215

[B117] Piccinini M. Rinaudo M. T. Chiapello N. Ricotti E. Baldovino S. Mostert M. (2002). The Human 26S Proteasome Is a Target of Antiretroviral Agents. Aids 16 (5), 693–700. 10.1097/00002030-200203290-00004 11964525

[B118] Purvis J. E. Karhohs K. W. Mock C. Batchelor E. Loewer A. Lahav G. (2012). p53 Dynamics Control Cell Fate. Science 336 (6087), 1440–1444. 10.1126/science.1218351 22700930 PMC4162876

[B119] Qiu Y. Maione F. Capano S. Meda C. Picconi O. Brundu S. (2020). HIV Protease Inhibitors Block HPV16-Induced Murine Cervical Carcinoma and Promote Vessel Normalization in Association with MMP-9 Inhibition and TIMP-3 Induction. Mol. Cancer Ther. 19 (12), 2476–2489. 10.1158/1535-7163.mct-20-0055 33082275

[B120] Rajagopalan S. Jaulent A. M. Wells M. Veprintsev D. B. Fersht A. R. (2008). 14-3-3 Activation of DNA Binding of P53 by Enhancing its Association into Tetramers. Nucleic Acids Res. 36 (18), 5983–5991. 10.1093/nar/gkn598 18812399 PMC2566891

[B121] Rajagopalan S. Sade R. S. Townsley F. M. Fersht A. R. (2010). Mechanistic Differences in the Transcriptional Activation of P53 by 14-3-3 Isoforms. Nucleic Acids Res. 38 (3), 893–906. 10.1093/nar/gkp1041 19933256 PMC2817464

[B122] Rasi Bonab F. Baghbanzadeh A. Ghaseminia M. Bolandi N. Mokhtarzadeh A. Amini M. (2021). Molecular Pathways in the Development of HPV-Induced Cervical Cancer. EXCLI J. 20, 320–337. 10.17179/excli2021-3365 33746665 PMC7975633

[B123] Rauschenbach L. Wieland A. Reinartz R. Kebir S. Till A. Darkwah Oppong M. (2020). Drug Repositioning of Antiretroviral Ritonavir for Combinatorial Therapy in Glioblastoma. Eur. J. Cancer 140, 130–139. 10.1016/j.ejca.2020.09.017 33091717

[B124] Rengan R. Mick R. Pryma D. A. Lin L. L. Christodouleas J. Plastaras J. P. (2019). Clinical Outcomes of the HIV Protease Inhibitor Nelfinavir with Concurrent Chemoradiotherapy for Unresectable Stage IIIA/IIIB Non-small Cell Lung Cancer. JAMA Oncol. 5 (10), 1464–1472. 10.1001/jamaoncol.2019.2095 31436839 PMC6707020

[B125] Roberts N. A. Martin J. A. Kinchington D. Broadhurst A. V. Craig J. C. Duncan I. B. (1990). Rational Design of Peptide-Based HIV Proteinase Inhibitors. Science 248 (4953), 358–361. 10.1126/science.2183354 2183354

[B126] Rodon J. Dienstmann R. Serra V. Tabernero J. (2013). Development of PI3K Inhibitors: Lessons Learned from Early Clinical Trials. Nat. Rev. Clin. Oncol. 10 (3), 143–153. 10.1038/nrclinonc.2013.10 23400000

[B127] Saidu N. E. B. Filić V. Thomas M. Sarabia-Vega V. Đukić A. Miljković F. (2019). PDZ Domain-Containing Protein NHERF-2 Is a Novel Target of Human Papillomavirus 16 (HPV-16) and HPV-18. J. Virol. 94 (1), e00663–19. 10.1128/JVI.00663-19 31597772 PMC6912117

[B194] Sahasrabuddhe V. V. Mwanahamuntu M. H. Vermund S. H. Huh W. K. Lyon M. D. Stringer J. S. A. (2007). Prevalence and Distribution of HPV Genotypes Among HIV-Infected Women in Zambia. British J.Cancer 96(9), 1480–1483. 10.1038/sj.bjc.6603737 PMC2360194 17437020

[B128] Schmitt A. M. Garcia J. T. Hung T. Flynn R. A. Shen Y. Qu K. (2016). An Inducible Long Noncoding RNA Amplifies DNA Damage Signaling. Nat. Genet. 48 (11), 1370–1376. 10.1038/ng.3673 27668660 PMC5083181

[B129] Sgadari C. Monini P. Barillari G. Ensoli B. (2003). Use of HIV Protease Inhibitors to Block Kaposi's Sarcoma and Tumour Growth. Lancet Oncol. 4 (9), 537–547. 10.1016/s1470-2045(03)01192-6 12965274

[B130] Sgadari C. Bacigalupo I. Barillari G. Ensoli B. (2011). Pharmacological Management of Kaposi's Sarcoma. Expert Opin. Pharmacother. 12 (11), 1669–1690. 10.1517/14656566.2011.577066 21517697

[B131] Sharma S. Mandal P. Sadhukhan T. Roy Chowdhury R. Ranjan Mondal N. Chakravarty B. (2015). Bridging Links between Long Noncoding RNA HOTAIR and HPV Oncoprotein E7 in Cervical Cancer Pathogenesis. Sci. Rep. 5 (1), 11724–11815. 10.1038/srep11724 26152361 PMC4495428

[B132] Sharma S. Munger K. (2018). Expression of the Cervical Carcinoma Expressed PCNA Regulatory (CCEPR) Long Noncoding RNA Is Driven by the Human Papillomavirus E6 Protein and Modulates Cell Proliferation Independent of PCNA. Virology 518, 8–13. 10.1016/j.virol.2018.01.031 29427865 PMC5911229

[B133] Sharma S. Munger K. (2020). The Role of Long Noncoding RNAs in Human Papillomavirus-Associated Pathogenesis. Pathogens 9 (4), 289. 10.3390/pathogens9040289 PMC7238103 32326624

[B134] Shiels M. S. Engels E. A. (2017). Evolving Epidemiology of HIV-Associated Malignancies. Curr. Opin. HIV AIDS 12 (1), 6–11. 10.1097/coh.0000000000000327 27749369 PMC5240042

[B135] Shim J. S. Rao R. Beebe K. Neckers L. Han I. Nahta R. (2012). Selective Inhibition of HER2-Positive Breast Cancer Cells by the HIV Protease Inhibitor Nelfinavir. J. Natl. Cancer Inst. 104 (20), 1576–1590. 10.1093/jnci/djs396 23042933 PMC3472971

[B136] Shmakova A. Germini D. Vassetzky Y. (2020). HIV‐1, HAART and Cancer: A Complex Relationship. Int. J. Cancer 146 (10), 2666–2679. 10.1002/ijc.32730 31603989

[B192] Shrestha S. Sudenga S. L. Smith J. S. Bachmann L. H. Wilson Kempf M. C. (2010). The Impact Of Highly Active Antiretroviral Therapy On Prevalence And Incidence Of Cervical Human Papillomavirus Infections in HIV-Positive Adolescents. BMC Infect. Dis. 10(1), 1–11. 10.1186/1471-2334-10-295 20946655 PMC2965148

[B137] Sias C. Salichos L. Lapa D. Del Nonno F. Baiocchini A. Capobianchi M. R. (2019). Alpha, Beta, Gamma Human PapillomaViruses (HPV) Detection with a Different Sets of Primers in Oropharyngeal Swabs, Anal and Cervical Samples. Virol. J. 16 (1), 27–10. 10.1186/s12985-019-1132-x 30832688 PMC6398256

[B138] Sinha T. (2018). Tumors: Benign and Malignant. Cancer Ther. Oncol. Int. J. 10 (3), 52–54. 10.19080/ctoij.2018.10.555790

[B139] Sombogaard F. Franssen E. J. F. Terpstra W. E. Kerver E. D. van den Berk G. E. L. Crul M. (2018). Outcome Effects of Antiretroviral Drug Combinations in HIV-Positive Patients with Chemotherapy for Lymphoma: a Retrospective Analysis. Int. J. Clin. Pharm. 40 (5), 1402–1408. 10.1007/s11096-018-0620-1 29948741 PMC6208603

[B140] Soprano M. Sorriento D. Rusciano M. R. Maione A. S. Limite G. Forestieri P. (2016). Oxidative Stress Mediates the Antiproliferative Effects of Nelfinavir in Breast Cancer Cells. PloS one 11 (6), e0155970. 10.1371/journal.pone.0155970 27280849 PMC4900679

[B141] Sousa H. Santos A. M. Pinto D. Medeiros R. (2011). Is There a Biological Plausability for P53 Codon 72 Polymorphism Influence on Cervical Cancer Development? Acta Med. Port 24 (1), 127–134. 21672450

[B142] Spugnini E. P. Esposito V. Groeger A. M. Cassandro R. Onori N. Chirianni A. (2006). Effects of Indinavir in a Preliminary Phase I Study on Dogs with Stage III Slenic Hemangiosarcoma. In Vivo 20 (1), 125–127. 16433040

[B143] Srirangam A. Milani M. Mitra R. Guo Z. Rodriguez M. Kathuria H. (2011). The Human Immunodeficiency Virus Protease Inhibitor Ritonavir Inhibits Lung Cancer Cells, in Part, by Inhibition of Survivin. J. Thorac. Oncol. 6 (4), 661–670. 10.1097/jto.0b013e31820c9e3c 21270666 PMC3104055

[B144] Srirangam A. Mitra R. Wang M. Gorski J. C. Badve S. Baldridge L. (2006). Effects of HIV Protease Inhibitor Ritonavir on Akt-Regulated Cell Proliferation in Breast Cancer. Clin. Cancer Res. 12 (6), 1883–1896. 10.1158/1078-0432.ccr-05-1167 16551874 PMC2727652

[B145] Stelzle D. Tanaka L. F. Lee K. K. Ibrahim Khalil A. Baussano I. Shah A. S. V. (2021). Estimates of the Global burden of Cervical Cancer Associated with HIV. Lancet Glob. Health 9 (2), e161–e169. 10.1016/s2214-109x(20)30459-9 33212031 PMC7815633

[B146] Stuqui B. Conceição A. L. Termini L. Sichero L. Villa L. L. Rahal P. (2016). The Differential Role of HTRA1 in HPV-Positive and HPV-Negative Cervical Cell Line Proliferation. BMC cancer 16 (1), 840–848. 10.1186/s12885-016-2873-1 27809811 PMC5095955

[B147] Subbaiah V. K. Kranjec C. Thomas M. Banks L. (2011). PDZ Domains: the Building Blocks Regulating Tumorigenesis. Biochem. J. 439 (2), 195–205. 10.1042/bj20110903 21954943

[B148] Subeha M. R. Telleria C. M. (2020). The Anti-cancer Properties of the HIV Protease Inhibitor Nelfinavir. Cancers 12 (11), 3437. 10.3390/cancers12113437 PMC7699465 33228205

[B149] Sun L. Niu L. Zhu X. Hao J. Wang P. Wang H. (2012). Antitumour Effects of a Protease Inhibitor, Nelfinavir, in Hepatocellular Carcinoma Cancer Cells. J. Chemother. 24 (3), 161–166. 10.1179/1973947812y.0000000011 22759761

[B150] Sung H. Ferlay J. Siegel R. L. Laversanne M. Soerjomataram I. Jemal A. (2021). Global Cancer Statistics 2020: GLOBOCAN Estimates of Incidence and Mortality Worldwide for 36 Cancers in 185 Countries. CA A. Cancer J. Clin. 71 (3), 209–249. 10.3322/caac.21660 33538338

[B151] Talis A. L. Huibregtse J. M. Howley P. M. (1998). The Role of E6AP in the Regulation of P53 Protein Levels in Human Papillomavirus (HPV)-positive and HPV-Negative Cells. J. Biol. Chem. 273 (11), 6439–6445. 10.1074/jbc.273.11.6439 9497376

[B152] Tanaka H. Arakawa H. Yamaguchi T. Shiraishi K. Fukuda S. Matsui K. (2000). A Ribonucleotide Reductase Gene Involved in a P53-dependent Cell-Cycle Checkpoint for DNA Damage. Nature 404 (6773), 42–49. 10.1038/35003506 10716435

[B153] Thomas M. Laura R. Hepner K. Guccione E. Sawyers C. Lasky L. (2002). Oncogenic Human Papillomavirus E6 Proteins Target the MAGI-2 and MAGI-3 Proteins for Degradation. Oncogene 21 (33), 5088–5096. 10.1038/sj.onc.1205668 12140759

[B154] Tommasino M. (2017). The Biology of Beta Human Papillomaviruses. Virus. Res. 231, 128–138. 10.1016/j.virusres.2016.11.013 27856220

[B155] Toschi E. Sgadari C. Malavasi L. Bacigalupo I. Chiozzini C. Carlei D. (2011). Human Immunodeficiency Virus Protease Inhibitors Reduce the Growth of Human Tumors via a Proteasome-independent Block of Angiogenesis and Matrix Metalloproteinases. Int. J. Cancer 128 (1), 82–93. 10.1002/ijc.25550 20617515

[B156] Travé G. Zanier K. (2016). HPV-mediated Inactivation of Tumor Suppressor P53. Cell Cycle 15 (17), 2231–2232. 10.1080/15384101.2016.1191257 27245825 PMC5004678

[B157] Tripathi V. Kaur E. Kharat S. S. Hussain M. Damodaran A. P. Kulshrestha S. (2019). Abrogation of FBW7α-dependent P53 Degradation Enhances P53's Function as a Tumor Suppressor. J. Biol. Chem. 294 (36), 13224–13232. 10.1074/jbc.ac119.008483 31346036 PMC6737220

[B158] Underbrink M. P. Howie H. L. Bedard K. M. Koop J. I. Galloway D. A. (2008). E6 Proteins from Multiple Human Betapapillomavirus Types Degrade Bak and Protect Keratinocytes from Apoptosis after UVB Irradiation. J. Virol. 82 (21), 10408–10417. 10.1128/jvi.00902-08 18715924 PMC2573196

[B159] Vats A. Skrabar N. Del Sal G. Banks L. (2022). Loss of the E6AP Ubiquitin Ligase Induces P53-dependent Phosphorylation of HPV-18 E6 in Cells Derived from Cervical Cancer. J. Virol. 96, e0150321. 10.1128/JVI.01503-21 35044207 PMC8941904

[B160] Vella S. (1994). Update on a Proteinase Inhibitor. Aids 8, S25–S30. 10.1097/00002030-199409001-00006 7840913

[B161] Voshavar C. (2019). Protease Inhibitors for the Treatment of HIV/AIDS: Recent Advances and Future Challenges. Ctmc 19 (18), 1571–1598. 10.2174/1568026619666190619115243 31237209

[B162] Wang Y.-W. Zhang K. Zhao S. Lv Y. Zhu J. Liu H. (2017). HPV Status and its Correlation with BCL2, P21, P53, Rb, and Survivin Expression in Breast Cancer in a Chinese Population. Biomed. Research International 2017, 1–7. 10.1155/2017/6315392 PMC5750508 29423411

[B163] Wee P. Wang Z. (2017). Epidermal Growth Factor Receptor Cell Proliferation Signaling Pathways. Cancers 9 (5), 52. 10.3390/cancers9050052 PMC5447962 28513565

[B164] Wei Q. (2005). Pitx2a Binds to Human Papillomavirus Type 18 E6 Protein and Inhibits E6-Mediated P53 Degradation in HeLa Cells. J. Biol. Chem. 280 (45), 37790–37797. 10.1074/jbc.m502974200 16129685 PMC1479768

[B165] Wen X. Li D. Zhang Y. Liu S. Ghali L. Iles R. K. (2012). Arsenic Trioxide Induces Cervical Cancer Apoptosis, but Specifically Targets Human Papillomavirus-Infected Cell Populations. Anti-Cancer Drugs 23 (3), 280–287. 10.1097/cad.0b013e32834f1fd3 22245994

[B166] White E. A. Kramer R. E. Tan M. J. A. Hayes S. D. Harper J. W. Howley P. M. (2012). Comprehensive Analysis of Host Cellular Interactions with Human Papillomavirus E6 Proteins Identifies New E6 Binding Partners and Reflects Viral Diversity. J. Virol. 86 (24), 13174–13186. 10.1128/jvi.02172-12 23015706 PMC3503137

[B167] Wu W. J. Shen Y. Sui J. Li C. Y. Yang S. Xu S. Y. (2018). Integrated Analysis of Long Non-coding RNA C-ompeting I-nteractions R-evealed P-otential B-iomarkers in C-ervical C-ancer: Based on a P-ublic D-atabase. Mol. Med. Rep. 17 (6), 7845–7858. 10.3892/mmr.2018.8846 29620291

[B168] Xia C. Chen R. Chen J. Qi Q. Pan Y. Du L. (2017). Combining Metformin and Nelfinavir Exhibits Synergistic Effects against the Growth of Human Cervical Cancer Cells and Xenograft in Nude Mice. Sci. Rep. 7 (1), 43373–43413. 10.1038/srep43373 28252027 PMC5333097

[B169] Xiang T. Du L. Pham P. Zhu B. Jiang S. (2015). Nelfinavir, an HIV Protease Inhibitor, Induces Apoptosis and Cell Cycle Arrest in Human Cervical Cancer Cells via the ROS-dependent Mitochondrial Pathway. Cancer Lett. 364 (1), 79–88. 10.1016/j.canlet.2015.04.027 25937300

[B170] Xie X. Piao L. Bullock B. N. Smith A. Su T. Zhang M. (2014). Targeting HPV16 E6-P300 Interaction Reactivates P53 and Inhibits the Tumorigenicity of HPV-Positive Head and Neck Squamous Cell Carcinoma. Oncogene 33 (8), 1037–1046. 10.1038/onc.2013.25 23474763 PMC3912227

[B171] Yang H. Nkeze J. Zhao R. Y. (2012). Effects of HIV-1 Protease on Cellular Functions and Their Potential Applications in Antiretroviral Therapy. Cell Biosci 2 (1), 32–38. 10.1186/2045-3701-2-32 22971934 PMC3490751

[B172] Yang H.-Y. Wen Y.-Y. Chen C.-H. Lozano G. Lee M.-H. (2003). 14-3-3σ Positively Regulates P53 and Suppresses Tumor Growth. Mol. Cel Biol 23 (20), 7096–7107. 10.1128/mcb.23.20.7096-7107.2003 PMC230310 14517281

[B173] Yang Y. Ikezoe T. Nishioka C. Bandobashi K. Takeuchi T. Adachi Y. (2006). NFV, an HIV-1 Protease Inhibitor, Induces Growth Arrest, Reduced Akt Signalling, Apoptosis and Docetaxel Sensitisation in NSCLC Cell Lines. Br. J. Cancer 95 (12), 1653–1662. 10.1038/sj.bjc.6603435 17133272 PMC2360758

[B174] Yilmaz G. Biswas-Fiss E. E. Biswas S. B. (2018). Genetic Variations in the DNA Replication Origins of Human Papillomavirus Family Correlate with Their Oncogenic Potential. Biochim. Biophys. Acta (Bba) - Gen. Subjects 1862 (4), 979–990. 10.1016/j.bbagen.2017.12.010 29288769

[B175] Yoshimatsu Y. Nakahara T. Tanaka K. Inagawa Y. Narisawa-Saito M. Yugawa T. (2017). Roles of the PDZ-Binding Motif of HPV 16 E6 Protein in Oncogenic Transformation of Human Cervical Keratinocytes. Cancer Sci. 108 (7), 1303–1309. 10.1111/cas.13264 28440909 PMC5497797

[B176] Yuan C.-H. Filippova M. Duerksen-Hughes P. (2012). Modulation of Apoptotic Pathways by Human Papillomaviruses (HPV): Mechanisms and Implications for Therapy. Viruses 4 (12), 3831–3850. 10.3390/v4123831 23250450 PMC3528293

[B177] Zhang J. Gao Y. (2019). Long Non-coding RNA MEG3 Inhibits Cervical Cancer Cell Growth by Promoting Degradation of P-STAT3 Protein via Ubiquitination. Cancer Cel Int 19 (1), 175–210. 10.1186/s12935-019-0893-z PMC6615085 31320837

[B178] Zhang P. Elabd S. Hammer S. Solozobova V. Yan H. Bartel F. (2015). TRIM25 Has a Dual Function in the p53/Mdm2 Circuit. Oncogene 34 (46), 5729–5738. 10.1038/onc.2015.21 25728675

[B179] Zhao C. Y. Szekely L. Bao W. Selivanova G. (2010). Rescue of P53 Function by Small-Molecule RITA in Cervical Carcinoma by Blocking E6-Mediated Degradation. Cancer Res. 70 (8), 3372–3381. 10.1158/0008-5472.can-09-2787 20395210

[B180] Zhou J. Du T. Li B. Rong Y. Verkhratsky A. Peng L. (2015). Crosstalk between MAPK/ERK and PI3K/AKT Signal Pathways during Brain Ischemia/reperfusion. ASN neuro 7 (5), 1759091415602463. 10.1177/1759091415602463 26442853 PMC4601130

[B181] Zhou Y. Zhong Y. Wang Y. Zhang X. Batista D. L. Gejman R. (2007). Activation of P53 by MEG3 Non-coding RNA. J. Biol. Chem. 282 (34), 24731–24742. 10.1074/jbc.m702029200 17569660

[B182] Zhu H. Chen X. Hu Y. Shi Z. Zhou Q. Zheng J. (2017). Long Non-coding RNA Expression Profile in Cervical Cancer Tissues. Oncol. Lett. 14 (2), 1379–1386. 10.3892/ol.2017.6319 28789353 PMC5529948

[B183] Zimmermann M. Koreck A. Meyer N. Basinski T. Meiler F. Simone B. (2011). TNF-like Weak Inducer of Apoptosis (TWEAK) and TNF-α Cooperate in the Induction of Keratinocyte Apoptosis. J. Allergy Clin. Immunol. 127 (1), 200–207. 10.1016/j.jaci.2010.11.005 21211655

[B184] zur Hausen H. (1977). Human Papillomaviruses and Their Possible Role in Squamous Cell Carcinomas. Curr. Top. Microbiol. Immunol. 78, 1–30. 10.1007/978-3-642-66800-5_1 202434

