# Supplementary figures and images for: Molecular Mechanisms of HIV Protease Inhibitors Against HPV-Associated Cervical Cancer: Restoration of TP53 Tumour Suppressor Activities
Source: Front Mol Biosci. 2022 May 10;9:875208. doi: 10.3389/fmolb.2022.875208 (PMC9127998; doi:10.3389/fmolb.2022.875208)

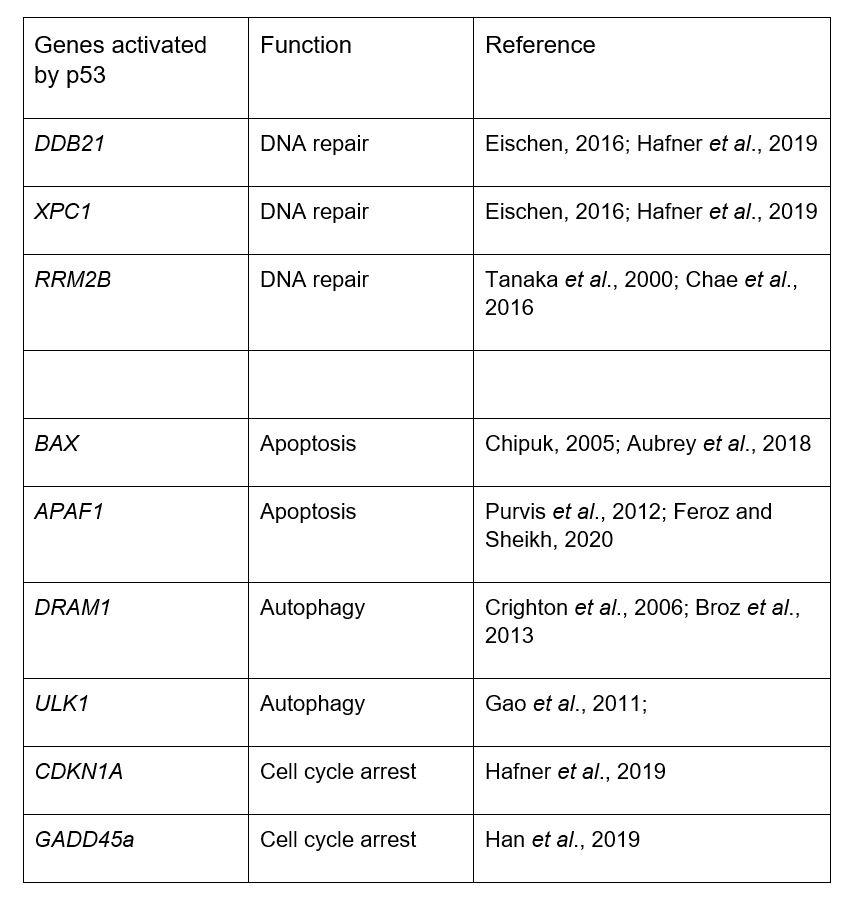

Supplement: Supplementary file 1 [file Image1.JPEG]

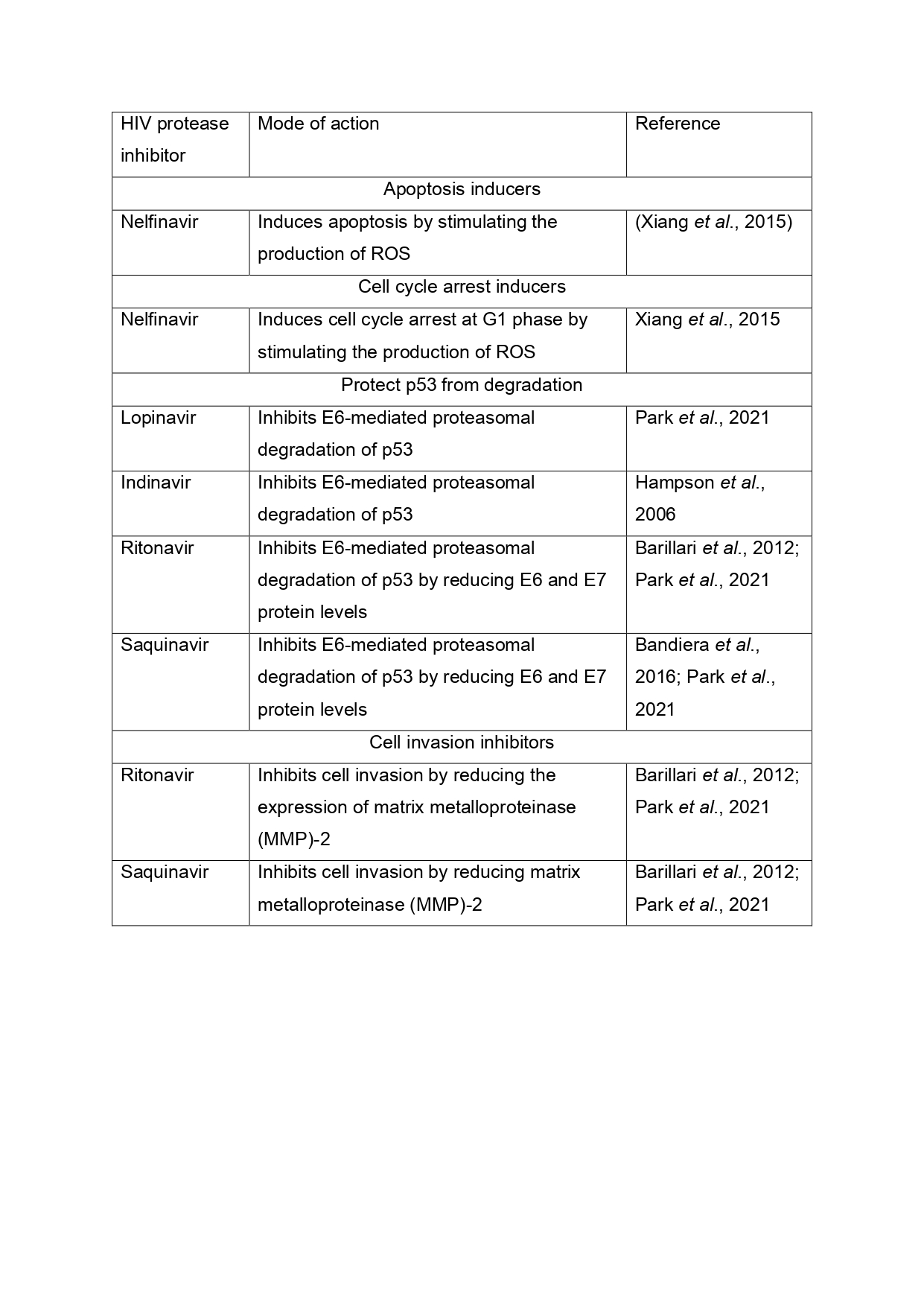

Supplement: Supplementary file 2 [file Image2.JPEG]
